# Clinical prognostic models in children with sepsis in low- and middle-income countries: a systematic review and meta-analysis

**DOI:** 10.3389/fped.2024.1463986

**Published:** 2024-10-17

**Authors:** Jessica Jordan, Celinie M. Nguyen, Lauren M. Fletcher, Stephanie C. Garbern

**Affiliations:** ^1^ Warren Alpert Medical School, Brown University, Providence, RI, United States; ^2^ Brown University Library, Brown University, Providence, RI, United States; ^3^ Department of Emergency Medicine, Warren Alpert Medical School, Brown University, Providence, RI, United States

**Keywords:** global health, emergency medicine, pediatrics, sepsis, low-and middle-income countries, prognostic model

## Abstract

**Introduction:**

Sepsis is the leading cause of child death worldwide, with the majority of these deaths occurring in low- and middle-income countries (LMICs). The aim of this systematic review and meta-analysis was to describe clinical prognostic scores and models for pediatric sepsis outcomes and assess the performance of these scores for predicting mortality in LMICs.

**Methods:**

Ovid Medline, CINAHL, Cochrane Library, EBSCO Global Health, and Web of Science, were searched through September 2022 for citations related to the development or validation of a clinical prognostic score or model among children with sepsis, conducted in LMIC. Titles, abstracts, and full texts were screened by two independent reviewers and data extracted included population characteristics, variables included, outcomes, and model performance. Risk of bias was assessed with the Prediction Model Risk of Bias Assessment Tool (PROBAST).

**Results:**

4,251 titles/abstracts and 315 full-text studies were screened, with 12 studies meeting inclusion criteria. Study countries included India, China, Egypt, Indonesia, Tanzania, and a multi-site study in Latin America. Prognostic scores/models included existing scores such as PELOD-2, pSOFA, PRISM, P-MODS, refractory shock criteria. There was high risk of bias in all studies. Meta-analysis was possible for pSOFA, PELOD-2, PRISM, and P-MODS, with pooled area under the receiver-operator characteristic curve of 0.86 (95%CI 0.78–0.94), 0.83 (95% CI 0.76–0.91), respectively.

**Conclusion:**

Relatively few clinical scores and models have been externally validated for prognostication and risk-stratification among children with sepsis in diverse LMIC settings. Notably there were no studies from low-income countries. Some potentially relevant studies were excluded due to lack of clarity regarding the presence of sepsis in the study populations. More widespread and standardized use of sepsis criteria may aid in better understanding the burden of sepsis and prognostic model performance at the bedside among children in LMICs. Further research to externally validate, implement and adapt these models is needed to account for challenges in use of these scores in resource-limited settings.

**Systematic Review Registration:**

https://www.crd.york.ac.uk/prospero/display_record.php?ID=CRD42022340126, PROSPERO [CRD42022340126].

## 1 Introduction

Sepsis is defined as life-threatening organ dysfunction caused by a dysregulated host response to infection and is frequently the common pathway to death from many infectious diseases (1). An estimated 25 million children experienced sepsis in 2017, leading to more than 3 million deaths (2). Low-and middle-income countries (LMICs) bear a disproportionate burden of global childhood deaths related to sepsis from acute infections such as pneumonia, diarrhea and malaria (3). In many LMICs, pediatric mortality and morbidity from sepsis remains exceptionally high for a multitude of reasons including inadequate critical care infrastructure, shortages of trained healthcare workers, higher levels of co-infections and malnutrition, as well as late identification of sepsis severity. Early recognition and risk stratification of sepsis remains pivotal for clinical management and preventing mortality (4). However, the recognition and management of sepsis remains challenging particularly in LMICs due to a lack of diagnostic and prognostic tools developed or validated for use in resource-constrained settings.

From 2005 to 2024, definitions for pediatric sepsis were based on the 2005 International Pediatric Sepsis Consensus Conference, where sepsis was defined as having two or more systemic inflammatory response syndrome (SIRS) criteria in the setting of confirmed or suspected infection, with severe sepsis denoting sepsis complicated by organ failure, and septic shock indicating sepsis with severe cardiovascular dysfunction (5). In January 2024, the International Consensus Criteria for Pediatric Sepsis and Septic Shock released updated definition in which sepsis is defined as 2 or more points in the Phoenix sepsis score indicating potentially life-threatening organ dysfunction of the respiratory, cardiovascular, coagulation, and/or neurological systems in children with suspected or confirmed infection (6). This long-awaited update to the pediatric sepsis criteria was developed to align with organ-failure based 2016 adult Sepsis-3 criteria, which use the Sequential Organ Failure Assessment (SOFA) score to confirm sepsis (1). As the Sepsis-3 criteria were not adapted for pediatric use for many years after Sepsis-3, multiple pediatric scoring systems had been proposed to help diagnose and risk-stratify pediatric sepsis, including the Pediatric Sequential Organ Failure Assessment (pSOFA) (7), the Pediatric Logistic Organ Dysfunction (PELOD/PELOD-2) score (Leteurtre 2003, 2013), and the Pediatric Multiple Organ Dysfunction (P-MODS) score (8).

However, studies validating these scores and other clinical prognostic models among children with sepsis in LMICs remain limited, and many prognostic scores and models rely on advanced laboratory testing usually unavailable in LMIC clinical environments. Evaluating the performance of existing sepsis prognostic scores to predict clinical outcomes in pediatric populations in LMICs is critical to understanding which of these tools is most feasible and applicable for use in a wide variety of LMIC contexts, to allow clinicians in these settings to make better-informed decisions for patient care. The aim of this review was to evaluate the performance of sepsis prognostic scores or models in pediatric populations in LMICs.

## 2 Materials and methods

This systematic review was conducted according to the Preferred Reporting Items for Systematic Reviews and Meta-analyses (PRISMA), with further guidance from Damen et al. on systematic reviews and meta-analysis for prognostic models (9). The review was registered on PROSPERO (registration number CRD42022340126) on July 26, 2022 (10). As only published, de-identified data were used, this study was exempt from institutional review board approval.

### 2.1 Search strategy and data sources

A medical librarian (LMF) developed an initial search string in Medline (Ovid) utilizing keywords related to sepsis, prognostic instruments, and a Lower and Middle Income Country (LMIC) filter developed by a collaboration of the Cochrane Effective Practice and Organization of Care Group, the WHO Library, and the Campbell Collaboration last updated in December 2022. The initial search was commented on and edited by the author team and then translated to the appropriate database syntax by the medical librarian. Final searches were conducted on September 6, 2022 in Medline (Ovid), Embase (Elsevier), CINAHL (EBSCO), Global Health (EBSCO), Web of Science (Clarivate), and Cochrane CENTRAL (Wiley). All references were imported to EndNote 20 and deduplicated. Deduplicated references were then imported into Covidence Systematic Review software. The search strings are included in Supplementary Materials.

### 2.2 Inclusion criteria

The review question was framed using the PICOTS format:

• Population: Pediatric patients (2 months–17 years) with sepsis
• Index model: All available prognostic scores or models
• Comparator model: Not applicable
• Outcome: All clinical outcomes (e.g., mortality, hospital length-of-stay, etc.)
• Timing: Prediction is at time of sepsis diagnosis or admission
• Setting: Any low- or middle-income country

Eligible studies included all clinical studies that evaluated a clinical model or score used for prognostication of sepsis in-patient mortality or relevant clinical outcome (mortality, hospital length-of-stay, ICU admission, use of mechanical ventilation, etc.), conducted primarily among children (<18 years old), in an LMIC using 2022 World Bank classifications. Models which included only biomarkers or only advanced laboratory tests not commonly found in LMICs (microRNA, etc.) were excluded given their limited utility among many LMIC settings which was the goal of this review. There were no exclusions made based on the etiology of sepsis (bacterial/viral/fungal, organ system source, etc.). If sepsis was not explicitly used as inclusion criteria in the study, the authors used pediatric SIRS-based sepsis criteria (SIRS criteria plus suspected infection) to make an assessment to the best of their ability as to whether the majority of the children in the study likely met the definition of sepsis (using 2022 criteria), with final determination agreed upon by consensus among the authors. SIRS criteria include: Temperature <36 degrees, >38 degrees; heart rate >90 beats per min; respiratory rate >20 breaths per min; WBC > 4,000 cells per mm >12, 000 cells per mm, immature band forms. If insufficient information was presented to determine if the study population focused on children with sepsis, the article was excluded.

### 2.3 Study selection

Two reviewers (JJ and CMN) independently screened each title and abstract, with discrepancies resolved by SCG. The same procedure was followed for full-text screening. Articles were excluded if they were not in English, were irrelevant to the topic (not focused primarily on children with sepsis), did not evaluate a model or score used for mortality prognostication, were not undertaken in an LMIC, or only consisted of an abstract. Studies that focused on evaluation of scores/models for use in detecting/diagnosing sepsis or for evaluation of multiple organ dysfunction syndrome that did not include assessment of mortality risk were excluded. The reference lists of similar reviews were searched manually to both verify search sensitivity and identify other potentially relevant studies.

### 2.4 Assessment of risk of bias

Risk of Bias was completed using the Prediction model study Risk Of Bias Assessment Tool (PROBAST) according to four domains as previously described in the literature: study participants, predictors, outcome, and analysis (11). Each domain has a series of questions to identify any areas of bias. If any domains were flagged with a high likelihood of bias, then the study was deemed as having high overall bias as per PROBAST guidelines. Two reviewers individually performed this assessment, and used discussion to come to consensus for each article, with discrepancies resolved by a third author (SCG). Cohen's kappa coefficient was determined to measure the interrater reliability.

### 2.5 Data extraction and analysis

Two authors independently evaluated each article and extracted data on a standardized form. A third author resolved any discrepancies. Form fields included the author, title, publication date, study country, World Bank country classification, study setting, study design, model/score description, primary and secondary outcomes. A narrative synthesis was used to report characteristics of included scores/models for sepsis mortality prognostication.

### 2.6 Summary measures and meta-analysis

Performance measures for each studied model/score were extracted for the outcome of inpatient mortality, including model discrimination and calibration (if reported), and whether the study was from a development or validation cohort (or both). Discrimination refers to the ability of a prediction model to differentiate between those who do or do not experience the outcome event. It is commonly estimated by the area under the receiver operating characteristic curve (AUC or c-statistic) which reflects the probability that for any randomly selected pair of individuals, one with and one without the outcome, the model assigns a higher probability to the individual with the outcome. The c-index can range from 0 to 1, with 1 indicating perfect discriminative ability and 0.5 implying that the model's predictions have an equal chance. Calibration refers to the accuracy of the predicted risk probabilities, which indicates the agreement between estimated and observed number of events in a cohort. Calibration may be presented in a calibration plot (expected probabilities plotted against observed outcome frequencies), as ratios between observed and expected number of events or outcome frequencies (O:E ratios), or through the Hosmer-Lemeshow goodness-of-fit statistic.

While data were extracted for all included studies, including assessments of risk of bias, meta-analysis was only conducted for scores/models that had performance measures in two or more articles for the primary outcome of mortality. Measures of discrimination (AUC) and calibration (calibration slope or HL test) were extracted from the included studies, where reported. If the standard errors (SEs) were not reported, it was calculated as follows [SE = upper limit of 95% CI—the lower limit of 95% CI/(21.96)]. A weighted average of the prediction model's discrimination performance (AUC) was calculated, and the weighted average was defined by the SE and the sample size of each study. The I^2^ statistic was calculated to assess the degree of heterogeneity, with a value exceeding 50% implying substantial heterogeneity (12). If substantial heterogeneity was found (due to differences in population characteristics, study design, and data sources, etc.) a random effects meta-analysis was performed to summarize performance of prognostic scores/models using restricted maximum likelihood (REML) estimation with meta in Stata 16 (StataCorp, College Station, TX), with 95% prediction intervals. Forest plots were used to display AUCs of studies included in meta-analysis. Of note, Mianling et al. 2019 and Zhong et al. 2020 used the same patient cohort and same dataset and thus were included only once in the meta-analysis.

## 3 Results

### 3.1 Study selection and characteristics

**Figure 1:**
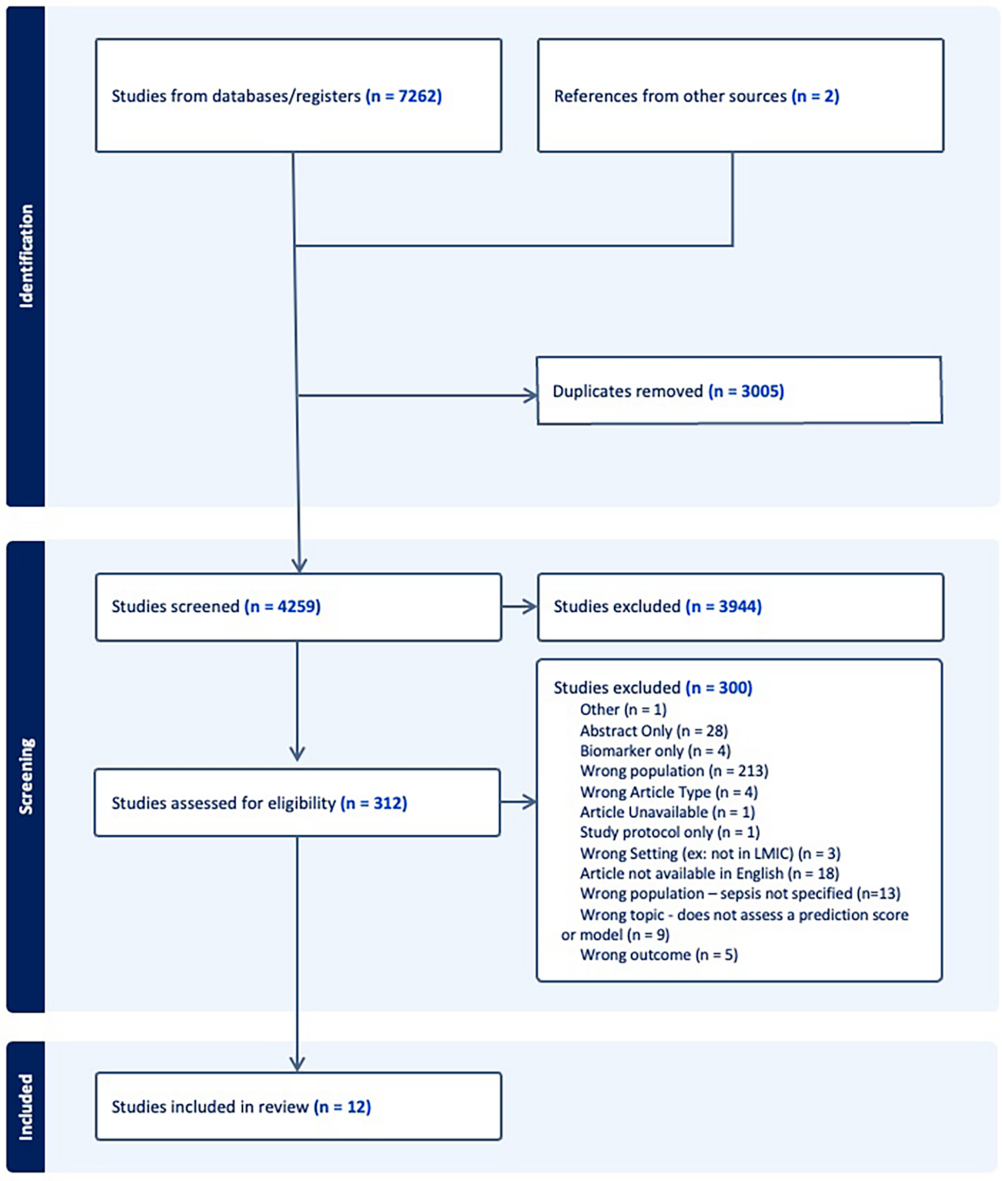
PRISMA flow diagram.

The search strategy retrieved 7,262 initial articles, after which 3,005 duplicate studies were removed and the remaining 4,259 articles were screened on title and abstract. The full texts of 312 studies were screened and 12 articles were included for analysis. The PRISMA flowchart in Figure 1 details the search process.

Study characteristics are shown in Supplementary Table S1. Six studies were conducted in lower-middle-income countries and five in upper-middle-income countries with one multi-country study in South America including a mix of country-income levels. Geographical spread included studies conducted in China, India, Egypt, Tanzania, Indonesia, a multi-country study (Brazil, Argentina, Chile, Paraguay, Ecuador). Of the included studies, there were 7 prospective cohort, 3 retrospective cohort, 2 *post-hoc* analysis of a prospective study or trial. Sample sizes ranged greatly from 60 to 1,831 patients. All but one study was conducted in a PICU setting. Inclusion criteria for what was considered pediatric shows some variation—the most common age groups were one month to either 14 or 17 years. Nearly all studies conducted a validation of a previously derived score or model. Only one study (Hu et al., 2016) developed a new model using a combination of clinical and laboratory markers. Clinical outcome measures included mortality (13), ICU length of stay, shock reversal, and need for mechanical ventilation. A narrative synthesis of each scoring model is detailed below.

### 3.2 Narrative synthesis and meta-analysis

#### 3.2.1 Pediatric sequential organ failure assessment (pSOFA)

**Figure 2:**
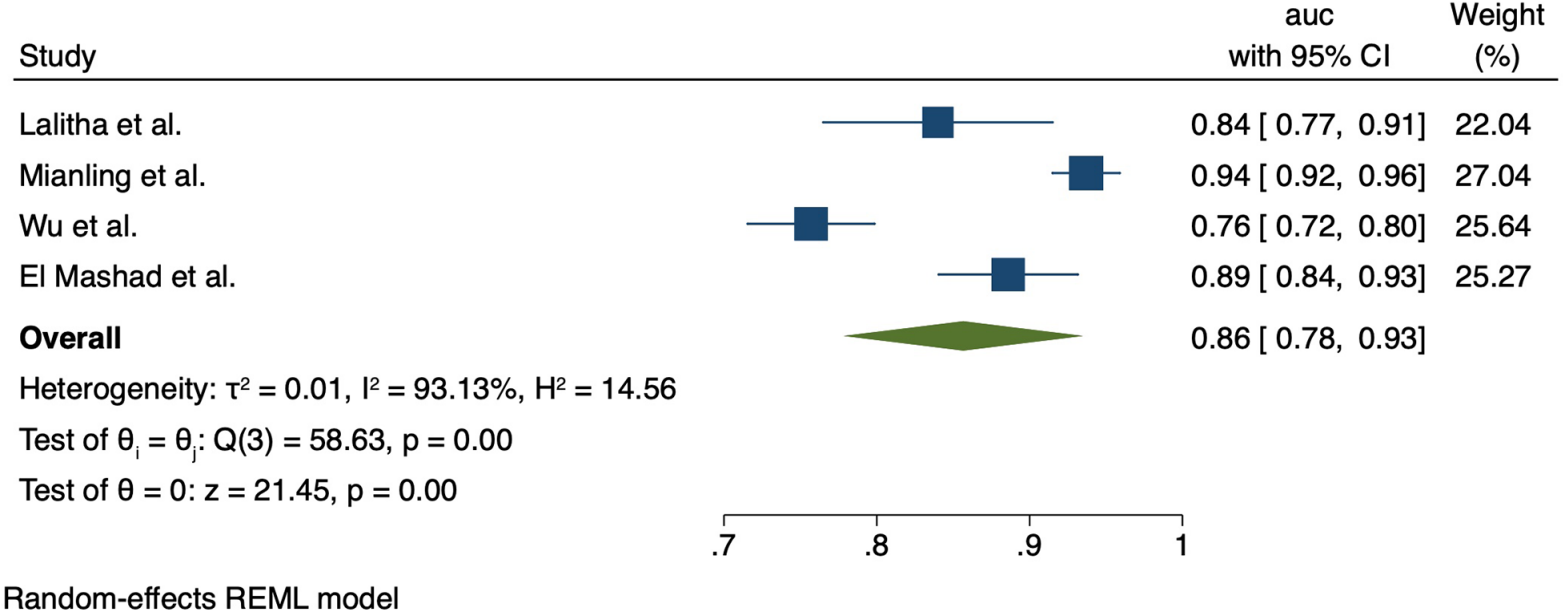
Forest plot for pSOFA AUC.

The pSOFA score was assessed in five studies; one in India (Lalitha et al. 2020), one in Egypt (El-Mashad) and three in China (Mianling et al. 2019, Wu et al. 2019, Zhong et al. 2020). The pSOFA showed strong performance for discrimination of mortality; in India (Lalitha et al. 2021), the AUC was 0.84 (95% CI 0.76–0.91). In the studies from China, AUCs were 0.937 (95% CI 0.913–0.957) (Mianling et al. 2019, Zhong et al. 2020), and 0.757 (99% CI 0.715–0.798) (Wu et al. 2019). In Egypt, the AUC was 0.89 (95% CI 0.84–0.931). Meta-analysis showed the pooled AUC for pSOFA for mortality was 0.86 (95% CI 0.78–0.93); heterogeneity *I*^2^ = 93.1%, *p* < 0.001). Forest plot for pSOFA pooled AUC is shown in Figure 2.

#### 3.2.2 Pediatric logistic organ dysfunction (PELOD and PELOD-2)

**Figure 3:**
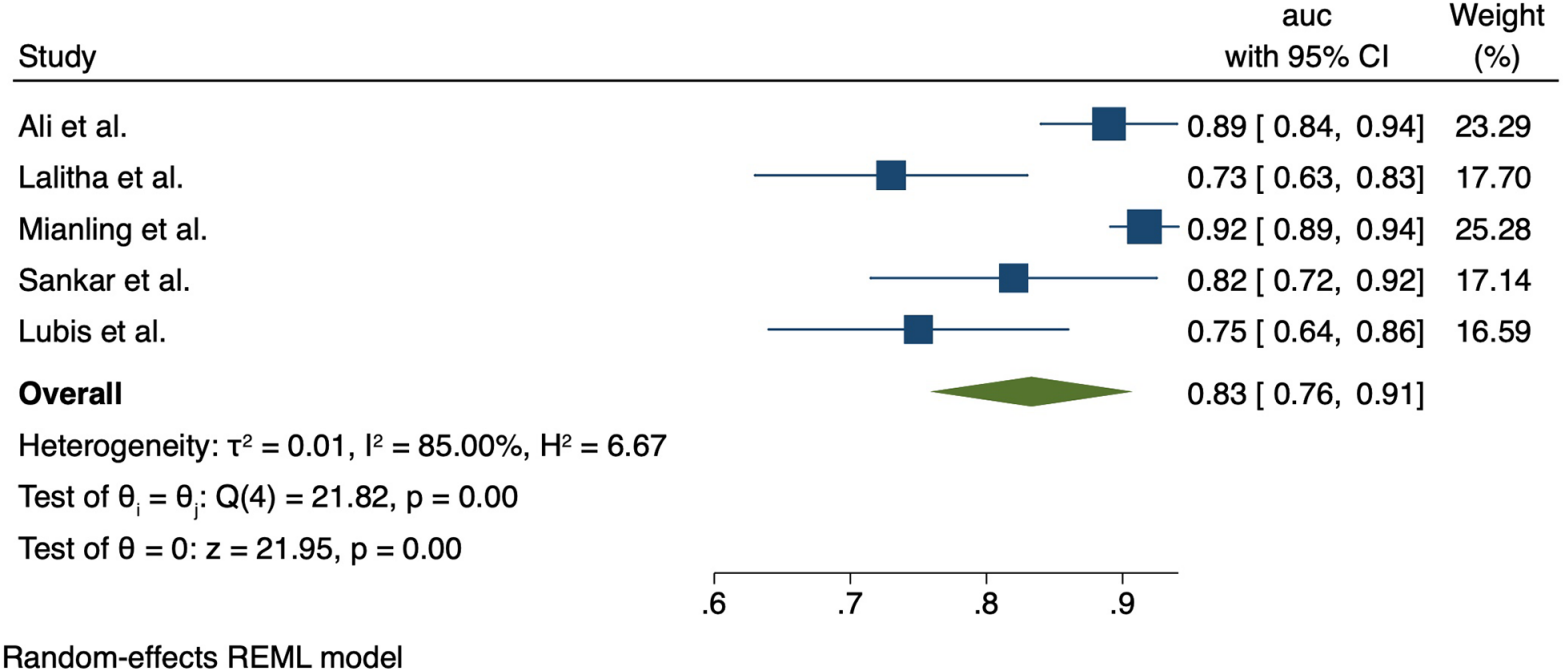
Forest plot for PELOD-2 AUC.

The PELOD-2 score was assessed in six studies; two in India (Ali et al. 2021, Lalitha et al. 2021), two in China (Zhong et al. 2020, Mianling et al. 2019), and two in Indonesia (Lubis et al. 2020, Yuniar 2021). The PELOD-2 reported AUCs were as follows: AUC 0.89 (0.84–0.94) (Ali et al. 2021), AUC 0.73 (95% CI 0.63–0.83) (Lalitha et al. 2021), (AUC, 0.916; 95% CI, 0.888–0.938) (Zhong et al. 2020, Mianling et al. 2019). In Indonesia, Lubis et al. found that addition of procalcitonin or C-reactive protein to the PELOD-2 score improved upon the performance of PELOD-2 alone, with the PELOD-2 + PCT performing the best (AUC 0.95 vs. PELOD-2 + CRP 0.80 vs. PELOD-2 alone 0.75). Meta-analysis showed the pooled AUC for PELOD-2 for mortality was 0.83 (95% CI 0.76–0.91); heterogeneity *I*^2^ = 85%, *p* < 0.001). Forest plot PELOD-2 is shown in Figure 3.

The PELOD (original) score was assessed in two prospective cohort studies (Souza et al. 2021, Thukral et al. 2007). Souza et al. 2021 studied the prevalence and outcomes of sepsis in pediatric populations in Brazil, Argentina, Chile, Paraguay and Ecuador, while Thukral et al. 2007 studied PICU patients in India. Souza et al. found PELOD was associated with greater mortality (OR 1.06, 95% CI 1.02–1.11) although model performance was not reported, while Thukral et al. found an AUC of 0.8 indicating good ability to discriminate death.

#### 3.2.3 Pediatric risk of mortality (PRISM)

The original PRISM score was assessed in two studies (Souza et al. 2021, El Mashad et al. 2020). One study compared data from Brazil, Argentina, Chile, Paraguay and Ecuador (Souza et al. 2021) while the other focused specifically on Egypt (El Mashad et al. 2020). Souza et al. found PRISM was associated with greater mortality (OR 1.06, 95% CI 1.02–1.11) although model performance was not reported, while El-Mashad et al. found an AUC = 0.79. Lalitha et al. 2021 studied the prognostic ability of the updated PRISM-III score as a prospective cohort in a PICU in India, and found AUC = 0.70 (95% CI = 0.61–0.8).

#### 3.2.4 Other models and scores

One study (Hu et al., 2016) aimed to develop a new scoring model to stratify pediatric sepsis severity using both clinical and laboratory values. Another study (Morin et al.) using the bSSS/cSSS to evaluate risk of mortality among children with refractory septic shock. P-MODS was evaluated in China (Zhong et al. 2020 and Mianling et al. 2019) in the same PICU cohort which AUC 0.761 (95% Ci 0.72–0.80).

### 3.3 Risk of bias/quality assessment

**Figure 4:**
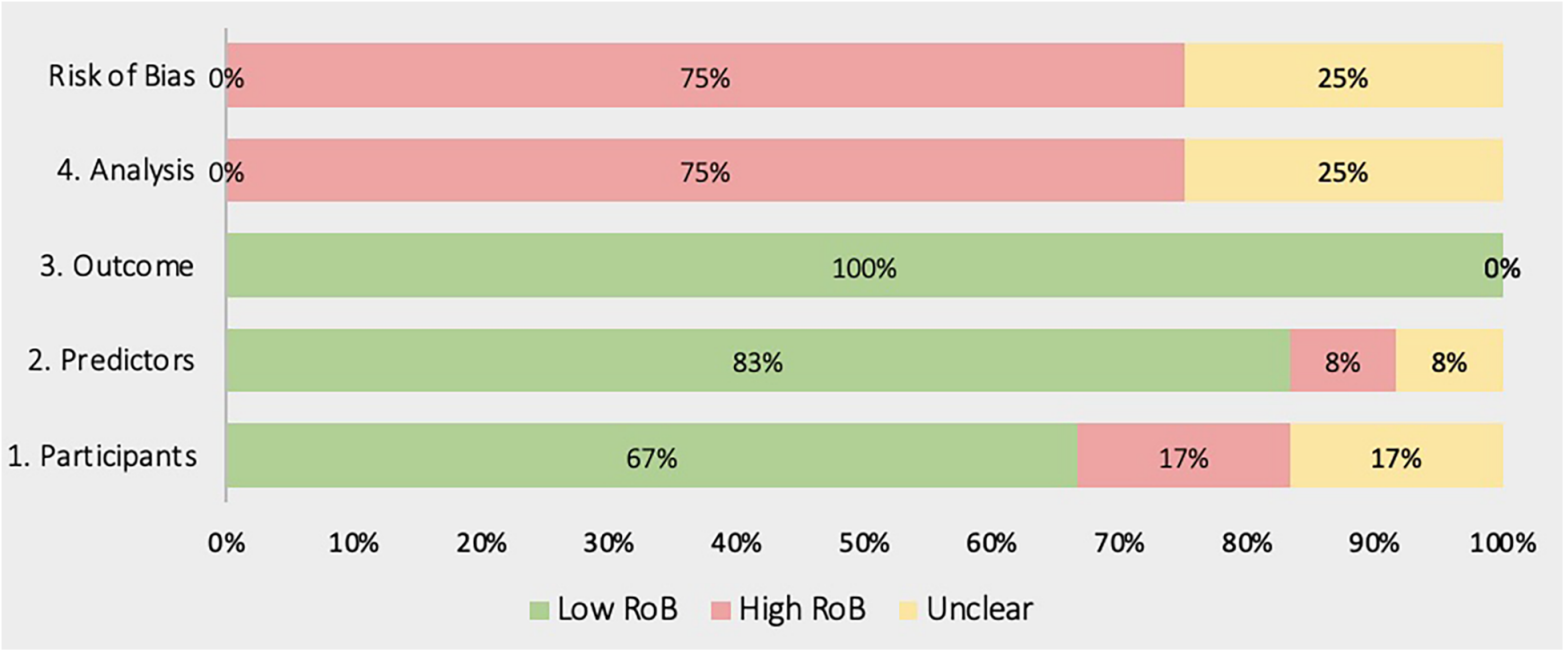
Summary risk of bias assessment using PROBAST.

**Figure 5:**
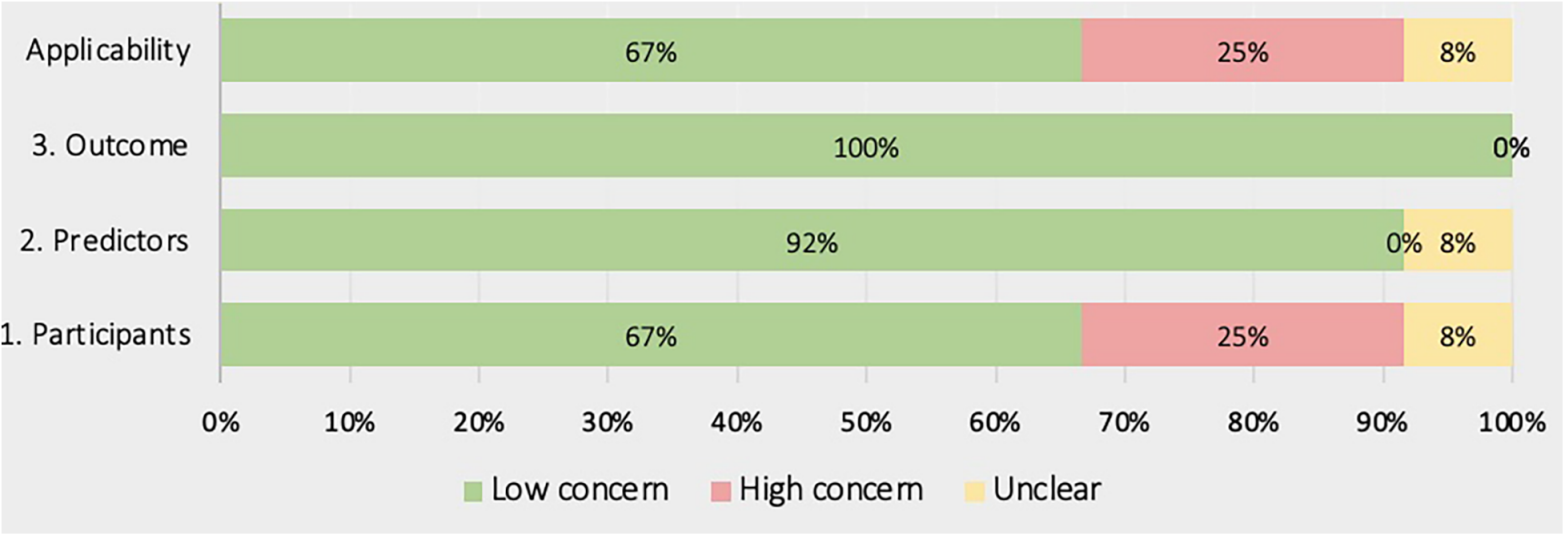
Summary of applicability assessment using PROBAST.

Figures 4, 5 show the overall risk of bias assessments and applicability assessment using PROBAST. The main sources of bias were in the analysis of studies with unclear or poor handling of missing data, insufficient reporting of performance measures particularly calibration measures. Additionally, there was often unclear or low applicability due to lack of sufficient information about the study population inclusion/exclusion criteria.

## 4 Discussion

Prognostic models and scores are valuable for clinicians to estimate a patient's risk of poor clinical outcomes and support informed clinical decision-making and resource allocation. This systematic review aimed to fill a gap in the literature regarding the performance of clinical prognostic models and scores specifically among septic children in LMICs. We identified 12 studies encompassing 15 different risk prediction scores or models, however only two scores or models (pSOFA, PELOD-2) were validated in two or more studies to allow meta-analysis. The pSOFA and PELOD-2 scores emerged as strong predictors of mortality in LMICs, with pooled AUCs 0.86 and 0.83, respectively, supporting their utility if resources allow for their calculation.

Validating prognostic scores and models specifically in LMICs is important when comparing prognostic performance in HICs vs. LMICs. Sun et al. (2022) conducted a meta-analysis on prognostic ability of pSOFA, SIRS, and qSOFA in pediatric populations with sepsis in HICs and LMICs (14). While there was only one HIC (Australia) included in that analysis, with pSOFA AUC of 0.83, the pSOFA AUC ranged from 0.75 to 0.84 in LMICs. Given the variation in score performance across individual studies in LMICs, this suggests a contextual dependence on prognostic ability and need for validation in diverse settings in both HICs and LMICs.

Several previous studies have explored the value of prognostic models for pediatric illnesses, although few have focused on the use of models in LMICs or focused specifically on sepsis which is the leading cause of child death (15, 16). Most recently, the Society of Critical Care Medicine (SCCM) redefined pediatric sepsis as based on the new Phoenix sepsis criteria (13). Although the Phoenix criteria accurately identified sepsis in data sets from lower-resourced settings, only 3.1% of the cohort used to validate the score came from low-resource settings. Additionally, all the low-resource validation sites had resources such as electronic health records and PICUs, which does not fully reflect conditions in many low-resource settings. This limits the generalizability of the new criteria by healthcare workers in low-resource settings (17). In the current literature, there have not been any external studies to date validating the Phoenix score in external settings. Future investigation of the Phoenix score in additional settings is imperative to assess its ability to be globally applicable, as intended to be by the international SCCM task force.

Van den Brink et al. also conducted a methodologically similar systematic review exploring risk prediction models in children in low-and middle-income countries (16). While the review was not focused on sepsis, they found the best performing models after meta-analysis to be SICK (ED), pSOFA and Pediatric Early Death Index for Africa (PEDIA)-immediate score (PW) and PELOD. The pSOFA score had a combined AUC of 0.86 in the PICU and pediatric wards, and the highest performing individual score in a PICU setting with an AUC of 0.94 across. They similarly found the PELOD-2 emerged as an effective tool for identifying children at risk of deterioration in resource-limited settings, with an AUC of 0.84 when exclusively used in the PICU. These findings are similar although less robust than the performance of pSOFA in its original validation study by Matics and Sanchez-Pinto (2017), where pSOFA had an AUC of 0.94 (7). Yuniar et al. (2023) conducted a scoping review to explore the quality and applicability of predictive models for determining pediatric sepsis mortality, focusing specifically on acute care and limited-resource settings (15) While no meta-analysis was conducted, and many models relied heavily on advanced laboratory markers, the final analysis included 28 mortality prediction models, and found that PRISM-III-APS, vasoactive-inotropic score at 12 h, albumin and lactate had excellent predictive values for mortality. Additionally, the PELOD-2 score emerged as a good mortality predictor, with an AUC of 0.916 on day of admission.

These results support the findings from the present systematic review, but also stress the importance of validating scores in lower resourced settings, and focusing on sepsis given the more standardized and improved ease of diagnosis with Phoenix criteria. Future research should focus on developing and validating prognostic scores among septic children as a unique population, and use standardized definitions for sepsis as inclusion criteria for studies. This will aid in developing clear recommendations for researchers in this space, while also guiding new prognostic scores and novel treatment interventions.

However, our findings highlight the lack of simple and well-validated prognostic scores and models, especially those developed specifically for use in resource-limited settings. We found only one study which developed and validated a new model (Hu et al. 2016 in China). Models specifically developed and externally validated within LMICs can help to ensure applicability across the broad spectrum of populations in LMICs. External validation of prognostic models is essential and it is imperative to use caution when applying these models in settings other than their original development context and instead to calibrate models to enhance their generalizability. This review also underscores the importance of increasing the diversity of LMIC representation within sepsis research globally.

In particular, there was a notable lack of studies from low-income countries and only one study from Latin America. Articles from urban hospitals in China and India made up the majority of included articles, which may limit the generalizability to more typical settings in LMICs broadly. Further research is needed in rural settings within LMICs, and in LICs where the burden of sepsis is often highest and also under-reported, and scarce resources exist for advanced critical care. The potential benefit of early risk stratification may thus be greatest in these settings.

Strengths of this review include a comprehensive search of databases related to pediatric global health, inclusive use of pediatric sepsis definitions, and use of the recently published PROBAST risk of bias tool. Limitations of this systematic review included the screening of only English language articles; inclusion of additional languages may increase the number of relevant articles available. Our inclusion criteria used a provisional diagnosis of sepsis for inclusion, however as sepsis is often not explicitly diagnosed due to difficulties in applying sepsis definitions in LMICs and not clearly reported in articles, this led to exclusion of some articles which likely included substantial proportion of children who were actually septic. Better reporting of sepsis, and use of sepsis criteria especially the new Phoenix criteria for sepsis diagnosis are greatly needed in LMICs.

Consistent with other recent reviews on related topics, many articles also had a high risk of bias, largely due to poor reporting of model performance, missingness, and selection bias. Bias can lead to over- or under-estimation of the ability of prognostic tools to correctly predict mortality in pediatric populations, thus it is essential to carefully consider bias in each study. Missingness of laboratory values is also of particular interest, as it is unclear from many of the articles whether there was significant missingness during calculation of the scores, which is a common issue in LMICs where laboratory tests are often not routine or are done at patients’ expense. Furthermore, some studies included laboratory biomarkers as predictors of mortality, and others focused on intensive care units, which are not always applicable in a lower-resourced setting, limiting the generalizability of those prognostic scores to all LMIC medical settings.

## 5 Conclusion

Relatively few clinical scores and models have been externally validated for prognostication and risk-stratification among children with sepsis in diverse LMIC settings. Based on our meta-analysis, pSOFA performed the best in predicting prognosis with a pooled AUC of 0.86 (CI) compared to the PELOD-2 with a pooled AUC of 0.83 (CI). A distinguishing advantage of the pSOFA score is the lower reliance on advanced laboratory diagnostics, making it more feasible in low-resource settings. Notably this review found no studies from low-income countries which met inclusion criteria. Some potentially relevant studies were excluded due to lack of clarity regarding the presence of sepsis in the study populations. More widespread and standardized use of sepsis criteria may aid in better understanding the burden of sepsis and prognostic model performance at the bedside among children in LMICs. Further research to externally validate, implement and adapt these models is needed to account for challenges in use of these scores in resource-limited settings. With the recent development of the Phoenix score, there is a necessity to investigate its performance within LMICs and low-resource settings to progress towards a more globally applicable and accurate prognostic score.

## Data Availability

The original contributions presented in the study are included in the article/Supplementary Material, further inquiries can be directed to the corresponding author.

## Supplementary material

Supplementary Table S1 Study characteristics and model performance.
