# Supplementary material for: Clinical prognostic models in children with sepsis in low- and middle-income countries: a systematic review and meta-analysis
Source: Front Pediatr. 2024 Oct 17;12:1463986. doi: 10.3389/fped.2024.1463986 (PMC11524808; doi:10.3389/fped.2024.1463986)
Supplement: Supplementary file 2 [file Table2.docx]

| Database | Medline |  |
| --- | --- | --- |
| Platform | Ovid |  |
| Date Late Searched | September 6, 2022 |  |
| Number of Results | 1882 |  |
| Notes |  |  |
| 1 | (Sepsis OR septicemia? OR blood poisoning? OR pyemia? OR pyohemia? OR bloodstream infection?).ti,ab,kf. | 139393 |
| 2 | exp sepsis/ | 137304 |
| 3 | Or/1-2 | 215190 |
| 4 | ((sepsis OR septic) adj3 (Prognos* OR algorithm OR tool? OR score? OR predictor? OR prediction OR calculator? OR index* OR diagnos* OR instrument? OR model? OR mortality OR "severity score" OR "mortality prediction" OR "risk stratification")).ti,ab,kf. | 20178 |
| 5 | "early warning score"/ | 332 |
| 6 | ((pediatric OR modified OR national) adj1 (early warning score)).ti,ab,kf. | 926 |
| 7 | ("PEWS" OR "EWS" OR "MEWS" OR "NEWS").ti,ab,kf. | 29532 |
| 8 | ("sequential organ failure assessment" OR "sofa" OR "qsofa" OR "pSOFA").ti,ab,kf. | 7627 |
| 9 | ("systemic inflammatory response syndrome" OR "sirs").ti,ab,kf. | 9041 |
| 10 | ("universal vital assessment" OR "UVA").ti,ab,kf. | 9073 |
| 11 | ("mortality in severe sepsis in the ED" OR "mortality in severe sepsis in the emergency department" OR "MISSED" OR "sMISSED").ti,ab,kf. | 55821 |
| 12 | ("risk-stratification of ED suspected sepsis" OR "risk-stratification of emergency department suspected sepsis" OR "REDS score").ti,ab,kf. | 2 |
| 13 | ("p?ediatric logistic organ dysfunction 2 score" OR PELOD-2 OR PELOD).ti,ab,kf. | 160 |
| 14 | ("neosep severity and recovery score*" OR "neosep severity score*" OR "neosep recovery score*").ti,ab,kf. | 0 |
| 15 | Or/4-14 | 128613 |
| 16 | (**afghanistan** OR **albania** OR **algeria** OR **american samoa** OR **angola** OR **"antigua and barbuda"** OR antigua OR barbuda OR **argentina** OR **armenia** OR armenian OR **aruba** OR **azerbaijan** OR **bahrain** OR **bangladesh** OR **barbados** OR **republic of belarus** OR belarus OR byelarus OR belorussia OR byelorussian OR **belize** OR british honduras OR **benin** OR dahomey OR **bhutan** OR **bolivia** OR **"bosnia and herzegovina"** OR Bosnia-Herzegovina OR bosnia OR herzegovina OR **botswana** OR bechuanaland OR **brazil** OR brasil OR **bulgaria** OR **burkina faso** OR burkina fasso OR upper volta OR **burundi** OR urundi OR **cabo verde** OR cape verde OR **cambodia** OR kampuchea OR khmer republic OR **cameroon** OR cameron OR cameroun OR **central african republic** OR ubangi shari OR **chad** OR **chile** OR **china** OR **colombia** OR **comoros** OR comoro islands OR iles comores OR mayotte OR **democratic republic of the congo** OR democratic republic congo OR **congo** OR zaire OR **costa rica** OR **"cote d’ivoire"** OR "cote d’ ivoire" OR cote divoire OR cote d ivoire OR ivory coast OR **croatia** OR **cuba** OR **cyprus** OR **czech republic** OR czechoslovakia OR **djibouti** OR french somaliland OR **dominica** OR **dominican republic** OR **ecuador** OR **egypt** OR united arab republic OR **el salvador** OR **equatorial guinea** OR spanish guinea OR **eritrea** OR **estonia** OR eswatini OR **swaziland** OR **ethiopia** OR **fiji** OR **gabon** OR gabonese republic OR **gambia** OR **"georgia (republic)"** OR georgian OR **ghana** OR gold coast OR **gibraltar** OR **greece** OR **grenada** OR **guam** OR **guatemala** OR **guinea** OR **guinea bissau** OR **guyana** OR british guiana OR **haiti** OR hispaniola OR **honduras** OR **hungary** OR **india** OR **indonesia** OR timor OR **iran** OR **iraq** OR isle of man OR **jamaica** OR **jordan** OR **kazakhstan** OR kazakh OR **kenya** OR "**democratic people’s republic of korea"** OR **republic of korea** OR north korea OR south korea OR korea OR **kosovo** OR **kyrgyzstan** OR kirghizia OR kirgizstan OR kyrgyz republic OR kirghiz OR **laos** OR lao pdr OR "lao people's democratic republic" OR **latvia** OR **lebanon** OR lebanese republic OR **lesotho** OR basutoland OR **liberia** OR **libya** OR libyan arab jamahiriya OR **lithuania** OR **macau** OR macao OR **republic of north macedonia** OR macedonia OR **madagascar** OR malagasy republic OR **malawi** OR nyasaland OR **malaysia** OR malay federation OR malaya federation OR maldives OR **indian ocean islands** OR indian ocean OR **mali** OR **malta** OR **micronesia** OR federated states of micronesia OR kiribati OR marshall islands OR nauru OR northern mariana islands OR **palau** OR tuvalu OR **mauritania** OR **mauritius** OR **mexico** OR **moldova** OR moldovian OR **mongolia** OR **montenegro** OR **morocco** OR ifni OR **mozambique** OR portuguese east africa OR **myanmar** OR burma OR **namibia** OR **nepal** OR **netherlands antilles** OR **nicaragua** OR **niger** OR **nigeria** OR **oman** OR muscat OR **pakistan** OR **panama** OR **papua new guinea** OR new guinea OR **paraguay** OR **peru** OR **philippines** OR philipines OR phillipines OR phillippines OR **poland** OR "polish people's republic" OR **portugal** OR portuguese republic OR **puerto rico** OR **romania** OR **russia** OR russian federation OR ussr OR soviet union OR union of soviet socialist republics OR **rwanda** OR ruanda OR **samoa** OR pacific islands OR polynesia OR samoan islands OR navigator island OR navigator islands OR **"sao tome and principe"** OR **saudi arabia** OR **senegal** OR **serbia** OR **seychelles** OR **sierra leone** OR **slovakia** OR slovak republic OR **slovenia** OR **melanesia** OR solomon island OR solomon islands OR norfolk island OR norfolk islands OR **somalia** OR **south africa** OR **south** **sudan** OR **sri lanka** OR ceylon OR **"saint kitts and nevis"** OR "st. kitts and nevis" OR **saint lucia** OR "st. lucia" OR **"saint vincent and the grenadines"** OR saint vincent OR "st. vincent" OR grenadines OR **sudan** OR **suriname** OR surinam OR dutch guiana OR netherlands guiana OR **syria** OR syrian arab republic OR **tajikistan** OR tadjikistan OR tadzhikistan OR tadzhik OR **tanzania** OR tanganyika OR **thailand** OR siam OR **timor leste** OR east timor OR **togo** OR togolese republic OR **tonga** OR **"trinidad and tobago"** OR trinidad OR tobago OR **tunisia** OR **turkey** OR **turkmenistan** OR turkmen OR **uganda** OR **ukraine** OR **uruguay** OR **uzbekistan** OR uzbek OR **vanuatu** OR new hebrides OR **venezuela** OR **vietnam** OR viet nam OR **middle east** OR west bank OR gaza OR palestine OR **yemen** OR **yugoslavia** OR **zambia** OR **zimbabwe** OR northern rhodesia OR global south OR **africa south of the sahara** OR sub-saharan africa OR subsaharan africa OR **africa, central** OR central africa OR **africa, northern** OR north africa OR northern africa OR magreb OR maghrib OR sahara OR **africa, southern** OR southern africa OR **africa, eastern** OR east africa OR eastern africa OR **africa, western** OR west africa OR western africa OR **west indies** OR **indian ocean islands** OR **caribbean** OR **central america** OR **latin america** OR "south and central america" OR **south america** OR **asia, central** OR central asia OR **asia, northern** OR north asia OR northern asia OR **asia, southeastern** OR southeastern asia OR south eastern asia OR southeast asia OR south east asia OR **asia, western** OR western asia OR **europe, eastern** OR east europe OR eastern europe OR developing country OR **developing countries** OR developing nation? OR developing population? OR developing world OR less developed countr* OR less developed nation? OR less developed population? OR less developed world OR lesser developed countr* OR lesser developed nation? OR lesser developed population? OR lesser developed world OR under developed countr* OR under developed nation? OR under developed population? OR under developed world OR underdeveloped countr* OR underdeveloped nation? OR underdeveloped population? OR underdeveloped world OR middle income countr* OR middle income nation? OR middle income population? OR low income countr* OR low income nation? OR low income population? OR lower income countr* OR lower income nation? OR lower income population? OR underserved countr* OR underserved nation? OR underserved population? OR underserved world OR under served countr* OR under served nation? OR under served population? OR under served world OR deprived countr* OR deprived nation? OR deprived population? OR deprived world OR poor countr* OR poor nation? OR poor population? OR poor world OR poorer countr* OR poorer nation? OR poorer population? OR poorer world OR developing econom* OR less developed econom* OR lesser developed econom* OR under developed econom* OR underdeveloped econom* OR middle income econom* OR low income econom* OR lower income econom* OR low gdp OR low gnp OR low gross domestic OR low gross national OR lower gdp OR lower gnp OR lower gross domestic OR lower gross national OR lmic OR lmics OR third world OR lami countr* OR transitional countr* OR emerging economies OR emerging nation?)**.ti,ab,sh,kf.** | 2264792 |
| 17 | 3 AND 15 AND 16 | 1,882 |
|  |  |  |

| Database | Embase |  |
| --- | --- | --- |
| Platform | Embase |  |
| Date Last Searched | September 6, 2022 |  |
| Number of Results | 1840 |  |
| Notes |  |  |
| 1 | (sepsis OR septicemia$ OR 'blood poisoning$' OR pyemia$ OR pyohemia$ OR 'bloodstream infection$'):ti,ab,kw | 213144 |
| 2 | 'sepsis'/exp | 319937 |
| 3 | #1 OR #2 | 378219 |
| 4 | ((sepsis OR septic) NEXT/3 (Prognos* OR algorithm OR tool$ OR score$ OR predictor$ OR prediction OR calculator$ OR index* OR diagnos* OR instrument$ OR model$ OR mortality OR 'severity score' OR 'mortality prediction' OR 'risk stratification')):ti,ab,kw | 13321 |
| 5 | 'early warning score'/exp | 1346 |
| 6 | ((p$ediatric OR modified OR national) NEXT/1 ('early warning score')):ti,ab,kw | 1499 |
| 7 | ('PEWS' OR 'EWS' OR 'MEWS' OR 'NEWS'):ti,ab,kw | 32830 |
| 8 | ('sequential organ failure assessment' OR 'sofa' OR 'qsofa' OR 'pSOFA'):ti,ab,kw | 15017 |
| 9 | ('systemic inflammatory response syndrome' OR 'sirs'):ti,ab,kw | 15223 |
| 10 | ('universal vital assessment' OR 'UVA'):ti,ab,kw | 16034 |
| 11 | ('mortality in severe sepsis in the ED' OR 'mortality in severe sepsis in the emergency department' OR 'MISSED' OR 'sMISSED'):ti,ab,kw | 87535 |
| 12 | ('risk-stratification of ED suspected sepsis' OR 'risk-stratification of emergency department suspected sepsis' OR 'REDS score'):ti,ab,kw | 6 |
| 13 | ('p$ediatric logistic organ dysfunction 2 score' OR 'PELOD-2' OR 'PELOD'):ti,ab,kw | 451 |
| 14 | ('neosep severity and recovery score*' OR 'neosep severity score*' OR 'neosep recovery score*'):ti,ab,kw | 1 |
| 15 | #4 OR #5 OR #6 OR #7 OR #8 OR #9 OR #10 OR #11 OR #12 OR #13 OR #14 | 177597 |
| 16 | (**afghanistan** OR **albania** OR **algeria** OR '**american samoa**' OR **angola** OR **antigua and barbuda** OR antigua OR barbuda OR **argentina** OR **armenia** OR armenian OR **aruba** OR **azerbaijan** OR **bahrain** OR **bangladesh** OR **barbados** OR **republic of belarus** OR belarus OR byelarus OR belorussia OR byelorussian OR **belize** OR british honduras OR **benin** OR dahomey OR **bhutan** OR **bolivia** OR **bosnia and herzegovina** OR Bosnia-Herzegovina OR bosnia OR herzegovina OR **botswana** OR bechuanaland OR **brazil** OR brasil OR **bulgaria** OR **burkina faso** OR burkina fasso OR upper volta OR **burundi** OR urundi OR '**cabo verde**' OR 'cape verde' OR **cambodia** OR kampuchea OR 'khmer republic' OR **cameroon** OR cameron OR cameroun OR '**central african republic**' OR 'ubangi shari' OR **chad** OR **chile** OR **china** OR **colombia** OR **comoros** OR 'comoro islands' OR 'iles comores' OR mayotte OR '**democratic republic of the congo**' OR 'democratic republic congo' OR **congo** OR zaire OR '**costa rica**' OR '**cote divoire**' OR 'cote d’ ivoire' OR 'cote divoire' OR 'cote d ivoire' OR 'ivory coast' OR **croatia** OR **cuba** OR **cyprus** OR '**czech republic**' OR czechoslovakia OR **djibouti** OR 'french somaliland' OR **dominica** OR '**dominican republic**' OR **ecuador** OR **egypt** OR 'united arab republic' OR '**el salvador**' OR '**equatorial guinea**' OR 'spanish guinea' OR **eritrea** OR **estonia** OR eswatini OR **swaziland** OR **ethiopia** OR **fiji** OR **gabon** OR 'gabonese republic' OR **gambia** OR **georgia (republic)** OR georgian OR **ghana** OR 'gold coast' OR **gibraltar** OR **greece** OR **grenada** OR **guam** OR **guatemala** OR **guinea** OR '**guinea bissau**' OR **guyana** OR 'british guiana' OR **haiti** OR hispaniola OR **honduras** OR **hungary** OR **india** OR **indonesia** OR timor OR **iran** OR **iraq** OR 'isle of man' OR **jamaica** OR **jordan** OR **kazakhstan** OR kazakh OR **kenya** OR '**democratic peoples republic of korea**' OR '**republic of korea**' OR 'north korea' OR 'south korea' OR korea OR **kosovo** OR **kyrgyzstan** OR kirghizia OR kirgizstan OR 'kyrgyz republic' OR kirghiz OR **laos** OR 'lao pdrv' OR 'lao peoples democratic republic' OR **latvia** OR **lebanon** OR 'lebanese republic' OR **lesotho** OR basutoland OR **liberia** OR **libya** OR 'libyan arab jamahiriya' OR **lithuania** OR **macau** OR macao OR '**republic of north macedonia**' OR macedonia OR **madagascar** OR 'malagasy republic' OR **malawi** OR nyasaland OR **malaysia** OR 'malay federation' OR 'malaya federation' OR maldives OR '**indian ocean islands**' OR 'indian ocean' OR **mali** OR **malta** OR **micronesia** OR 'federated states of micronesia' OR kiribati OR 'marshall islands' OR nauru OR 'northern mariana islands' OR **palau** OR tuvalu OR **mauritania** OR **mauritius** OR **mexico** OR **moldova** OR moldovian OR **mongolia** OR **montenegro** OR **morocco** OR ifni OR **mozambique** OR 'portuguese east africa' OR **myanmar** OR burma OR **namibia** OR **nepal** OR '**netherlands antilles**' OR **nicaragua** OR **niger** OR **nigeria** OR **oman** OR muscat OR **pakistan** OR **panama** OR '**papua new guinea**' OR 'new guinea' OR **paraguay** OR **peru** OR **philippines** OR philipines OR phillipines OR phillippines OR **poland** OR 'polish peoples republic' OR **portugal** OR 'portuguese republic' OR '**puerto rico**' OR **romania** OR **russia** OR 'russian federation' OR ussr OR 'soviet union' OR 'union of soviet socialist republics' OR **rwanda** OR ruanda OR **samoa** OR 'pacific islands' OR polynesia OR 'samoan islands' OR 'navigator island' OR 'navigator islands' OR '**sao tome and principe**' OR '**saudi arabia**' OR **senegal** OR **serbia** OR **seychelles** OR '**sierra leone**' OR **slovakia** OR 'slovak republic' OR **slovenia** OR **melanesia** OR 'solomon island' OR 'solomon islands' OR 'norfolk island' OR 'norfolk islands' OR **somalia** OR '**south africa**' OR '**south** **sudan**' OR '**sri lanka**' OR ceylon OR '**saint kitts and nevis**' OR 'st. kitts and nevis' OR '**saint lucia**' OR 'st. lucia' OR '**saint vincent and the grenadines**' OR 'saint vincent' OR 'st. vincent' OR grenadines OR **sudan** OR **suriname** OR surinam OR 'dutch guiana' OR 'netherlands guiana' OR **syria** OR 'syrian arab republic' OR **tajikistan** OR tadjikistan OR tadzhikistan OR tadzhik OR **tanzania** OR tanganyika OR **thailand** OR siam OR '**timor leste**' OR 'east timor' OR **togo** OR 'togolese republic' OR **tonga** OR '**trinidad and tobago**' OR trinidad OR tobago OR **tunisia** OR **turkey** OR **turkmenistan** OR turkmen OR **uganda** OR **ukraine** OR **uruguay** OR **uzbekistan** OR uzbek OR **vanuatu** OR 'new hebrides' OR **venezuela** OR **vietnam** OR 'viet nam' OR '**middle east**' OR 'west bank' OR gaza OR palestine OR **yemen** OR **yugoslavia** OR **zambia** OR **zimbabwe** OR 'northern rhodesia' OR 'global south' OR '**africa south of the sahara**' OR 'sub-saharan africa' OR 'subsaharan africa' OR **africa, central** OR 'central africa' OR **africa, northern** OR 'north africa' OR 'northern africa' OR magreb OR maghrib OR sahara OR **africa, southern** OR 'southern africa' OR **africa, eastern** OR 'east africa' OR 'eastern africa' OR **africa, western** OR 'west africa' OR 'western africa' OR '**west indies**' OR '**indian ocean islands**' OR **caribbean** OR '**central america**' OR '**latin america**' OR 'south and central america' OR '**south america**' OR **asia, central** OR 'central asia' OR **asia, northern** OR 'north asia' OR 'northern asia' OR **asia, southeastern** OR 'southeastern asia' OR 'south eastern asia' OR 'southeast asia' OR 'south east asia' OR **asia, western** OR 'western asia' OR **europe, eastern** OR 'east europe' OR 'eastern europe' OR 'developing country' OR '**developing countries**' OR 'developing nation$' OR 'developing population$' OR 'developing world' OR 'less developed countr*' OR 'less developed nation$' OR 'less developed population$' OR 'less developed world' OR 'lesser developed countr*' OR 'lesser developed nation$' OR 'lesser developed population$' OR 'lesser developed world' OR 'under developed countr*' OR 'under developed nation$' OR 'under developed population$' OR 'under developed world' OR 'underdeveloped countr*' OR 'underdeveloped nation$' OR 'underdeveloped population$' OR 'underdeveloped world' OR 'middle income countr*' OR 'middle income nation$' OR 'middle income population$' OR 'low income countr*' OR 'low income nation$' OR 'low income population$' OR 'lower income countr*' OR 'lower income nation$' OR 'lower income population$' OR 'underserved countr*' OR 'underserved nation$' OR 'underserved population$' OR 'underserved world' OR 'under served countr*' OR 'under served nation$' OR 'under served population$' OR 'under served world' OR 'deprived countr*' OR 'deprived nation$' OR 'deprived population$' OR 'deprived world' OR 'poor countr*' OR 'poor nation$' OR 'poor population$' OR 'poor world' OR 'poorer countr*' OR 'poorer nation$' OR 'poorer population$' OR 'poorer world' OR 'developing econom*' OR 'less developed econom*' OR 'lesser developed econom*' OR 'under developed econom*' OR 'underdeveloped econom*' OR 'middle income econom*' OR 'low income econom*' OR 'lower income econom*' OR 'low gdp' OR 'low gnp' OR 'low gross domestic' OR 'low gross national' OR 'lower gdp' OR 'lower gnp' OR 'lower gross domestic' OR 'lower gross national' OR lmic OR lmics OR 'third world' OR 'lami countr*' OR 'transitional countr*' OR 'emerging economies' OR 'emerging nation$'):ti,ab,kw,de | 2623150 |
| 17 | #3 AND #15 AND #16 | 1840 |
|  |  |  |

| Database | Cochrane CENTRAL |  |
| --- | --- | --- |
| Platform | Cochrane Library |  |
| Date Late Searched | September 6, 2022 |  |
| Number of Results | 272 |  |
| Notes |  |  |
| 1 | (Sepsis OR septicemia? OR blood poisoning? OR pyemia? OR pyohemia? OR bloodstream infection?):ti,ab,kw | 14971 |
| 2 | [mh sepsis] | 4970 |
| 3 | #1 OR #2 | 16957 |
| 4 | ((sepsis OR septic) NEAR/3 (Prognos* OR algorithm OR tool? OR score? OR predictor? OR prediction OR calculator? OR index* OR diagnos* OR instrument? OR model? OR mortality OR "severity score" OR "mortality prediction" OR "risk stratification")):ti,ab,kw | 2202 |
| 5 | [mh "early warning score"] | 6 |
| 6 | ((p?ediatric OR modified OR national) NEAR/1 (early warning score)):ti,ab,kw | 615 |
| 7 | ("PEWS" OR "EWS" OR "MEWS" OR "NEWS"):ti,ab,kw | 2985 |
| 8 | ("sequential organ failure assessment" OR "sofa" OR "qsofa" OR "pSOFA"):ti,ab,kw | 2057 |
| 9 | ("systemic inflammatory response syndrome" OR "sirs"):ti,ab,kw | 1533 |
| 10 | ("universal vital assessment" OR "UVA"):ti,ab,kw | 819 |
| 11 | ("mortality in severe sepsis in the ED" OR "mortality in severe sepsis in the emergency department" OR "MISSED" OR "sMISSED"):ti,ab,kw | 4988 |
| 12 | ("risk-stratification of ED suspected sepsis" OR "risk-stratification of emergency department suspected sepsis" OR "REDS score"):ti,ab,kw | 0 |
| 13 | ("p?ediatric logistic organ dysfunction 2 score" OR PELOD-2 OR PELOD):ti,ab,kw | 37 |
| 14 | ("neosep severity and recovery score*" OR "neosep severity score*" OR "neosep recovery score*"):ti,ab,kw | 1 |
| 15 | #4 OR #5 OR #6 OR #7 OR #8 OR #9 OR #10 OR #11 OR #12 OR #13 OR #14 | 14450 |
| 16 | (**afghanistan** OR **albania** OR **algeria** OR **"american samoa"** OR **angola** OR **"antigua and barbuda"** OR antigua OR barbuda OR **argentina** OR **armenia** OR armenian OR **aruba** OR **azerbaijan** OR **bahrain** OR **bangladesh** OR **barbados** OR **"republic of belarus"** OR belarus OR byelarus OR belorussia OR byelorussian OR **belize** OR "british honduras" OR **benin** OR dahomey OR **bhutan** OR **bolivia** OR **"bosnia and herzegovina"** OR Bosnia-Herzegovina OR bosnia OR herzegovina OR **botswana** OR bechuanaland OR **brazil** OR brasil OR **bulgaria** OR **"burkina faso"** OR "burkina fasso" OR "upper volta" OR **burundi** OR urundi OR **"cabo verde"** OR "cape verde" OR **cambodia** OR kampuchea OR "khmer republic" OR **cameroon** OR cameron OR cameroun OR **"central african republic"** OR "ubangi shari" OR **chad** OR **chile** OR **china** OR **colombia** OR **comoros** OR "comoro islands" OR "iles comores" OR mayotte OR **"democratic republic of the congo"** OR "democratic republic congo" OR **congo** OR zaire OR **"costa rica"** OR **"cote d’ivoire"** OR "cote d’ ivoire" OR "cote divoire" OR "cote d ivoire" OR "ivory coast" OR **croatia** OR **cuba** OR **cyprus** OR **"czech republic"** OR czechoslovakia OR **djibouti** OR "french somaliland" OR **dominica** OR **"dominican republic"** OR **ecuador** OR **egypt** OR "united arab republic" OR **"el salvador"** OR **"equatorial guinea"** OR "spanish guinea" OR **eritrea** OR **estonia** OR eswatini OR **swaziland** OR **ethiopia** OR **fiji** OR **gabon** OR "gabonese republic" OR **gambia** OR **"georgia (republic)"** OR georgia OR georgian OR **ghana** OR "gold coast" OR **gibraltar** OR **greece** OR **grenada** OR **guam** OR **guatemala** OR **guinea** OR **"guinea bissau"** OR **guyana** OR "british guiana" OR **haiti** OR hispaniola OR **honduras** OR **hungary** OR **india** OR **indonesia** OR timor OR **iran** OR **iraq** OR "isle of man" OR **jamaica** OR **jordan** OR **kazakhstan** OR kazakh OR **kenya** OR **"democratic people’s republic of korea"** OR **"republic of korea"** OR north korea OR south korea OR korea OR **kosovo** OR **kyrgyzstan** OR kirghizia OR kirgizstan OR "kyrgyz republic" OR kirghiz OR **laos** OR "lao pdr" OR "lao people's democratic republic" OR **latvia** OR **lebanon** OR "lebanese republic" OR **lesotho** OR basutoland OR **liberia** OR **libya** OR "libyan arab jamahiriya" OR **lithuania** OR **macau** OR macao OR **"republic of north macedonia"** OR macedonia OR **madagascar** OR "malagasy republic" OR **malawi** OR nyasaland OR **malaysia** OR "malay federation" OR "malaya federation" OR maldives OR **"indian ocean islands"** OR "indian ocean" OR **mali** OR **malta** OR **micronesia** OR "federated states of micronesia" OR kiribati OR "marshall islands" OR nauru OR "northern mariana islands" OR **palau** OR tuvalu OR **mauritania** OR **mauritius** OR **mexico** OR **moldova** OR moldovian OR **mongolia** OR **montenegro** OR **morocco** OR ifni OR **mozambique** OR "portuguese east africa" OR **myanmar** OR burma OR **namibia** OR **nepal** OR **"netherlands antilles"** OR **nicaragua** OR **niger** OR **nigeria** OR **oman** OR muscat OR **pakistan** OR **panama** OR "**papua new guinea"** OR **paraguay** OR **peru** OR **philippines** OR philipines OR phillipines OR phillippines OR **poland** OR "polish people's republic" OR **portugal** OR "portuguese republic" OR **"puerto rico"** OR **romania** OR **russia** OR "russian federation" OR ussr OR "soviet union" OR "union of soviet socialist republics" OR **rwanda** OR ruanda OR **samoa** OR "pacific islands" OR polynesia OR "samoan islands" OR "navigator island" OR "navigator islands" OR **"sao tome and principe"** OR **"saudi arabia"** OR **senegal** OR **serbia** OR **seychelles** OR **"sierra leone"** OR **slovakia** OR "slovak republic" OR **slovenia** OR **melanesia** OR "solomon island" OR "solomon islands" OR "norfolk island" OR "norfolk islands" OR **somalia** OR **"south africa"** OR **"south sudan"** OR **"sri lanka"** OR ceylon OR **"saint kitts and nevis"** OR "st. kitts and nevis" OR **"saint lucia"** OR "st. lucia" OR **"saint vincent and the grenadines"** OR "saint vincent" OR "st. vincent" OR grenadines OR **sudan** OR **suriname** OR surinam OR "dutch guiana" OR "netherlands guiana" OR **syria** OR "syrian arab republic" OR **tajikistan** OR tadjikistan OR tadzhikistan OR tadzhik OR **tanzania** OR tanganyika OR **thailand** OR siam OR **"timor leste"** OR "east timor" OR **togo** OR "togolese republic" OR **tonga** OR **"trinidad and tobago"** OR trinidad OR tobago OR **tunisia** OR **turkey** OR **turkmenistan** OR turkmen OR **uganda** OR **ukraine** OR **uruguay** OR **uzbekistan** OR uzbek OR **vanuatu** OR "new hebrides" OR **venezuela** OR **vietnam** OR "viet nam" OR **"middle east"** OR "west bank" OR gaza OR palestine OR **yemen** OR **yugoslavia** OR **zambia** OR **zimbabwe** OR "northern rhodesia" OR "global south" OR **"africa south of the sahara"** OR "sub saharan africa" OR "subsaharan africa" OR **"africa, central"** OR "central africa" OR **"africa, northern**$\boldsymbol{"}$ OR "north africa" OR "northern africa" OR magreb OR maghrib OR sahara OR **"africa, southern**$\boldsymbol{"}$ OR "southern africa" OR **"africa, eastern**$\boldsymbol{"}$ OR "east africa" OR "eastern africa" OR **"africa, western**$\boldsymbol{"}$ OR $"$west africa$"$ OR $"$western africa$"$ OR **"west indies"** OR **"indian ocean islands"** OR **caribbean** OR **"central america"** OR **"latin america"** OR "south and central america" OR **"south america"** OR **"asia, central"** OR "central asia" OR **"asia, northern"** OR "north asia" OR "northern asia" OR **"asia, southeastern"** OR "southeastern asia" OR "south eastern asia" OR "southeast asia" OR "south east asia" OR **"asia, western"** OR "western asia" OR **"europe, eastern"** OR "east europe" OR "eastern europe" OR "developing country" OR **"developing countries"** OR "developing nation" OR "developing nations" OR "developing population" OR "developing populations" OR "developing world" OR "less developed country" OR "less developed countries" OR "less developed nation" OR "less developed nations" OR "less developed population" OR "less developed populations" OR "less developed world" OR "lesser developed country" OR "lesser developed countries" OR "lesser developed nation" OR "lesser developed nations" OR "lesser developed population" OR "lesser developed populations" OR "lesser developed world" OR "under developed country" OR "under developed countries" OR "under developed nation" OR "under developed nations" OR "under developed population" OR "under developed populations" OR "under developed world" OR "underdeveloped country" OR "underdeveloped countries" OR "underdeveloped nation" OR "underdeveloped nations" OR "underdeveloped population" OR "underdeveloped populations" OR "underdeveloped world" OR "middle income country" OR "middle income countries" OR "middle income nation" OR "middle income nations" OR "middle income population" OR "middle income populations" OR "low income country" OR "low income countries" OR "low income nation" OR "low income nations" OR "low income population" OR "low income populations" OR "lower income country" OR "lower income countries" OR "lower income nation" OR "lower income nations" OR "lower income population" OR "lower income populations" OR "underserved country" OR "underserved countries" OR "underserved nation" OR "underserved nations" OR "underserved population" OR "underserved populations" OR "underserved world" OR "under served country" OR "under served countries" OR "under served nation" OR "under served nations" OR "under served population" OR "under served populations" OR "under served world" OR "deprived country" OR "deprived countries" OR "deprived nation" OR "deprived nations" OR "deprived population" OR "deprived populations" OR "deprived world" OR "poor country" OR "poor countries" OR "poor nation" OR "poor nations" OR "poor population" OR "poor populations" OR "poor world" OR "poorer country" OR "poorer countries" OR "poorer nation" OR "poorer nations" OR "poorer population" OR "poorer populations" OR "poorer world" OR "developing economy" OR "developing economies" OR "less developed economy" OR "less developed economies" OR "lesser developed economy" OR "lesser developed economies" OR "under developed economy" OR "under developed economies" OR "underdeveloped economy" OR "underdeveloped economies" OR "middle income economy" OR "middle income economies" OR "low income economy" OR "low income economies" OR "lower income economy" OR "lower income economies" OR "low gdp" OR "low gnp" OR "low gross domestic" OR "low gross national" OR "lower gdp" OR "lower gnp" OR "lower gross domestic" OR "lower gross national" OR lmic OR lmics OR "third world" OR "lami country" OR "lami countries" OR "transitional country" OR "transitional countries" OR "emerging economies" OR "emerging nation" OR "emerging nations"):**ti,ab,kw** | 112247 |
| 17 | #3 AND #15 AND #16 | 272 |
|  |  |  |

| Database | CINAHL |  |
| --- | --- | --- |
| Platform | Ebsco |  |
| Date Late Searched | September 6, 2022 |  |
| Number of Results | 503 |  |
| Notes |  |  |
| 1 | TI(Sepsis OR septicemia# OR "blood poisoning#" OR pyemia# OR pyohemia# OR "bloodstream infection#") | 15236 |
| 2 | AB(Sepsis OR septicemia# OR "blood poisoning#" OR pyemia# OR pyohemia# OR "bloodstream infection#") | 26651 |
| 3 | (MH "Sepsis+") | 30850 |
| 4 | S1 OR S2 OR S3 | 46627 |
| 5 | TI((sepsis OR septic) W3 (Prognos* OR algorithm OR tool# OR score# OR predictor# OR prediction OR calculator# OR index* OR diagnos* OR instrument# OR model# OR mortality OR "severity score" OR "mortality prediction" OR "risk stratification")) | 684 |
| 6 | AB((sepsis OR septic) W3 (Prognos* OR algorithm OR tool# OR score# OR predictor# OR prediction OR calculator# OR index* OR diagnos* OR instrument# OR model# OR mortality OR "severity score" OR "mortality prediction" OR "risk stratification")) | 2392 |
| 7 | (MH "early warning score") | 118 |
| 8 | TI((p#ediatric OR modified OR national) W1 ("early warning score")) | 257 |
| 9 | AB((p#ediatric OR modified OR national) W1 ("early warning score")) | 460 |
| 10 | TI("PEWS" OR "EWS" OR "MEWS" OR "NEWS") | 52710 |
| 11 | AB("PEWS" OR "EWS" OR "MEWS" OR "NEWS") | 19326 |
| 12 | TI("sequential organ failure assessment" OR "sofa" OR "qsofa" OR "pSOFA") | 434 |
| 13 | AB("sequential organ failure assessment" OR "sofa" OR "qsofa" OR "pSOFA") | 2350 |
| 14 | TI("systemic inflammatory response syndrome" OR "sirs") | 1233 |
| 15 | AB("systemic inflammatory response syndrome" OR "sirs") | 2101 |
| 16 | TI("universal vital assessment" OR "UVA") | 105 |
| 17 | AB("universal vital assessment" OR "UVA") | 495 |
| 18 | TI("mortality in severe sepsis in the ED" OR "mortality in severe sepsis in the emergency department" OR "MISSED" OR "sMISSED") | 4018 |
| 19 | AB("mortality in severe sepsis in the ED" OR "mortality in severe sepsis in the emergency department" OR "MISSED" OR "sMISSED") | 15538 |
| 20 | TI("risk-stratification of ED suspected sepsis" OR "risk-stratification of emergency department suspected sepsis" OR "REDS score") | 1 |
| 21 | AB("risk-stratification of ED suspected sepsis" OR "risk-stratification of emergency department suspected sepsis" OR "REDS score") | 35 |
| 22 | TI("p#ediatric logistic organ dysfunction 2 score" OR PELOD-2 OR PELOD) | 20 |
| 23 | AB("p#ediatric logistic organ dysfunction 2 score" OR PELOD-2 OR PELOD) | 62 |
| 24 | TI("neosep severity and recovery score*" OR "neosep severity score*" OR "neosep recovery score*") | 0 |
| 25 | AB("neosep severity and recovery score*" OR "neosep severity score*" OR "neosep recovery score*") | 0 |
| 26 | S5 OR S6 OR S7 OR S8 OR S9 OR S10 OR S11 OR S12 OR S13 OR S14 OR S15 OR S16 OR S17 OR S18 OR S19 OR S20 OR S21 OR S22 OR S23 OR S24 OR S25 | 94498 |
| 27 | TI(**afghanistan** OR **albania** OR **algeria** OR "**american samoa**" OR **angola** OR **"antigua and barbuda"** OR antigua OR barbuda OR **argentina** OR **armenia** OR armenian OR **aruba** OR **azerbaijan** OR **bahrain** OR **bangladesh** OR **barbados** OR "**republic of belarus**" OR belarus OR byelarus OR belorussia OR byelorussian OR **belize** OR "british honduras" OR **benin** OR dahomey OR **bhutan** OR **bolivia** OR **"bosnia and herzegovina"** OR Bosnia-Herzegovina OR bosnia OR herzegovina OR **botswana** OR bechuanaland OR **brazil** OR brasil OR **bulgaria** OR "**burkina faso**" OR "burkina fasso" OR "upper volta" OR **burundi** OR urundi OR "**cabo verde**" OR "cape verde" OR **cambodia** OR kampuchea OR "khmer republic" OR **cameroon** OR cameron OR cameroun OR "**central african republic**" OR "ubangi shari" OR **chad** OR **chile** OR **china** OR **colombia** OR **comoros** OR "comoro islands" OR "iles comores" OR mayotte OR "**democratic republic of the congo**" OR "democratic republic congo" OR **congo** OR zaire OR "**costa rica**" OR **"cote d’ivoire"** OR "cote d’ ivoire" OR "cote divoire" OR "cote d ivoire" OR "ivory coast" OR **croatia** OR **cuba** OR **cyprus** OR "**czech republic**" OR czechoslovakia OR **djibouti** OR "french somaliland" OR **dominica** OR "**dominican republic**" OR **ecuador** OR **egypt** OR "united arab republic" OR "**el salvador**" OR "**equatorial guinea**" OR "spanish guinea" OR **eritrea** OR **estonia** OR eswatini OR **swaziland** OR **ethiopia** OR **fiji** OR **gabon** OR "gabonese republic" OR **gambia** OR **"georgia (republic)"** OR georgian OR **ghana** OR "gold coast" OR **gibraltar** OR **greece** OR **grenada** OR **guam** OR **guatemala** OR **guinea** OR "**guinea bissau**" OR **guyana** OR "british guiana" OR **haiti** OR hispaniola OR **honduras** OR **hungary** OR **india** OR **indonesia** OR timor OR **iran** OR **iraq** OR "isle of man" OR **jamaica** OR **jordan** OR **kazakhstan** OR kazakh OR **kenya** OR "**democratic people’s republic of korea"** OR "**republic of korea**" OR "north korea" OR "south korea" OR korea OR **kosovo** OR **kyrgyzstan** OR kirghizia OR kirgizstan OR "kyrgyz republic" OR kirghiz OR **laos** OR "lao pdr" OR "lao people's democratic republic" OR **latvia** OR **lebanon** OR "lebanese republic" OR **lesotho** OR basutoland OR **liberia** OR **libya** OR "libyan arab jamahiriya" OR **lithuania** OR **macau** OR macao OR "**republic of north macedonia**" OR macedonia OR **madagascar** OR "malagasy republic" OR **malawi** OR nyasaland OR **malaysia** OR "malay federation" OR malaya federation OR maldives OR "**indian ocean islands**" OR "indian ocean" OR **mali** OR **malta** OR **micronesia** OR "federated states of micronesia" OR kiribati OR "marshall islands" OR nauru OR "northern mariana islands" OR **palau** OR tuvalu OR **mauritania** OR **mauritius** OR **mexico** OR **moldova** OR moldovian OR **mongolia** OR **montenegro** OR **morocco** OR ifni OR **mozambique** OR "portuguese east africa" OR **myanmar** OR burma OR **namibia** OR **nepal** OR "**netherlands antilles**" OR **nicaragua** OR **niger** OR **nigeria** OR **oman** OR muscat OR **pakistan** OR **panama** OR "**papua new guinea**" OR "new guinea" OR **paraguay** OR **peru** OR **philippines** OR philipines OR phillipines OR phillippines OR **poland** OR "polish people's republic" OR **portugal** OR portuguese republic OR "**puerto rico**" OR **romania** OR **russia** OR "russian federation" OR ussr OR "soviet union" OR "union of soviet socialist republics" OR **rwanda** OR ruanda OR **samoa** OR "pacific islands" OR polynesia OR "samoan islands" OR "navigator island" OR "navigator islands" OR **"sao tome and principe"** OR "**saudi arabia**" OR **senegal** OR **serbia** OR **seychelles** OR "**sierra leone**" OR **slovakia** OR "slovak republic" OR **slovenia** OR **melanesia** OR "solomon island" OR "solomon islands" OR "norfolk island" OR "norfolk islands" OR **somalia** OR "**south africa**" OR "**south** **sudan**" OR "**sri lanka**" OR ceylon OR **"saint kitts and nevis"** OR "st. kitts and nevis" OR "**saint lucia**" OR "st. lucia" OR **"saint vincent and the grenadines"** OR "saint vincent" OR "st. vincent" OR grenadines OR **sudan** OR **suriname** OR surinam OR "dutch guiana" OR "netherlands guiana" OR **syria** OR "syrian arab republic" OR **tajikistan** OR tadjikistan OR tadzhikistan OR tadzhik OR **tanzania** OR tanganyika OR **thailand** OR siam OR "**timor leste**" OR "east timor" OR **togo** OR "togolese republic" OR **tonga** OR **"trinidad and tobago"** OR trinidad OR tobago OR **tunisia** OR **turkey** OR **turkmenistan** OR turkmen OR **uganda** OR **ukraine** OR **uruguay** OR **uzbekistan** OR uzbek OR **vanuatu** OR "new hebrides" OR **venezuela** OR **vietnam** OR "viet nam" OR "**middle east**" OR "west bank" OR gaza OR palestine OR **yemen** OR **yugoslavia** OR **zambia** OR **zimbabwe** OR "northern rhodesia" OR "global south" OR "**africa south of the sahara**" OR "sub-saharan africa" OR "subsaharan africa" OR "**africa, central**" OR "central africa" OR "**africa, northern**" OR "north africa" OR "northern africa" OR magreb OR maghrib OR sahara OR "**africa, southern**" OR "southern africa" OR "**africa, eastern**" OR "east africa" OR "eastern africa" OR "**africa, western**" OR "west africa" OR "western africa" OR "**west indies**" OR "**indian ocean islands**" OR **caribbean** OR "**central america**" OR "**latin america**" OR "south and central america" OR "**south america**" OR "**asia, central**" OR "central asia" OR "**asia, northern**" OR "north asia" OR "northern asia" OR "**asia, southeastern**" OR "southeastern asia" OR "south eastern asia" OR "southeast asia" OR "south east asia" OR "**asia, western**" OR "western asia" OR "**europe, eastern**" OR "east europe" OR "eastern europe" OR "developing country" OR "**developing countries**" OR "developing nation#" OR "developing population#" OR "developing world" OR "less developed countr*" OR "less developed nation#" OR "less developed population#" OR "less developed world" OR "lesser developed countr*" OR "lesser developed nation#" OR "lesser developed population#" OR "lesser developed world" OR "under developed countr*" OR "under developed nation#" OR "under developed population#" OR "under developed world" OR "underdeveloped countr*" OR "underdeveloped nation#" OR "underdeveloped population#" OR "underdeveloped world" OR "middle income countr*" OR "middle income nation#" OR "middle income population#" OR "low income countr*" OR "low income nation#" OR "low income population#" OR "lower income countr*" OR "lower income nation#" OR "lower income population#" OR "underserved countr*" OR "underserved nation#" OR "underserved population#" OR "underserved world" OR "under served countr*" OR "under served nation#" OR "under served population#" OR "under served world" OR "deprived countr*" OR "deprived nation#" OR "deprived population#" OR "deprived world" OR "poor countr*" OR "poor nation#" OR "poor population#" OR "poor world" OR "poorer countr*" OR "poorer nation#" OR "poorer population#" OR "poorer world" OR "developing econom*" OR "less developed econom*" OR "lesser developed econom*" OR "under developed econom*" OR "underdeveloped econom*" OR "middle income econom*" OR "low income econom*" OR "lower income econom*" OR "low gdp" OR "low gnp" OR "low gross domestic" OR "low gross national" OR "lower gdp" OR "lower gnp" OR "lower gross domestic" OR "lower gross national" OR lmic OR lmics OR "third world" OR "lami countr*" OR "transitional countr*" OR "emerging economies" OR "emerging nation#") | 219473 |
| 28 | AB(**afghanistan** OR **albania** OR **algeria** OR "**american samoa**" OR **angola** OR **"antigua and barbuda"** OR antigua OR barbuda OR **argentina** OR **armenia** OR armenian OR **aruba** OR **azerbaijan** OR **bahrain** OR **bangladesh** OR **barbados** OR "**republic of belarus**" OR belarus OR byelarus OR belorussia OR byelorussian OR **belize** OR "british honduras" OR **benin** OR dahomey OR **bhutan** OR **bolivia** OR **"bosnia and herzegovina"** OR Bosnia-Herzegovina OR bosnia OR herzegovina OR **botswana** OR bechuanaland OR **brazil** OR brasil OR **bulgaria** OR "**burkina faso**" OR "burkina fasso" OR "upper volta" OR **burundi** OR urundi OR "**cabo verde**" OR "cape verde" OR **cambodia** OR kampuchea OR "khmer republic" OR **cameroon** OR cameron OR cameroun OR "**central african republic**" OR "ubangi shari" OR **chad** OR **chile** OR **china** OR **colombia** OR **comoros** OR "comoro islands" OR "iles comores" OR mayotte OR "**democratic republic of the congo**" OR "democratic republic congo" OR **congo** OR zaire OR "**costa rica**" OR **"cote d’ivoire"** OR "cote d’ ivoire" OR "cote divoire" OR "cote d ivoire" OR "ivory coast" OR **croatia** OR **cuba** OR **cyprus** OR "**czech republic**" OR czechoslovakia OR **djibouti** OR "french somaliland" OR **dominica** OR "**dominican republic**" OR **ecuador** OR **egypt** OR "united arab republic" OR "**el salvador**" OR "**equatorial guinea**" OR "spanish guinea" OR **eritrea** OR **estonia** OR eswatini OR **swaziland** OR **ethiopia** OR **fiji** OR **gabon** OR "gabonese republic" OR **gambia** OR **"georgia (republic)"** OR georgian OR **ghana** OR "gold coast" OR **gibraltar** OR **greece** OR **grenada** OR **guam** OR **guatemala** OR **guinea** OR "**guinea bissau**" OR **guyana** OR "british guiana" OR **haiti** OR hispaniola OR **honduras** OR **hungary** OR **india** OR **indonesia** OR timor OR **iran** OR **iraq** OR "isle of man" OR **jamaica** OR **jordan** OR **kazakhstan** OR kazakh OR **kenya** OR "**democratic people’s republic of korea"** OR "**republic of korea**" OR "north korea" OR "south korea" OR korea OR **kosovo** OR **kyrgyzstan** OR kirghizia OR kirgizstan OR "kyrgyz republic" OR kirghiz OR **laos** OR "lao pdr" OR "lao people's democratic republic" OR **latvia** OR **lebanon** OR "lebanese republic" OR **lesotho** OR basutoland OR **liberia** OR **libya** OR "libyan arab jamahiriya" OR **lithuania** OR **macau** OR macao OR "**republic of north macedonia**" OR macedonia OR **madagascar** OR "malagasy republic" OR **malawi** OR nyasaland OR **malaysia** OR "malay federation" OR malaya federation OR maldives OR "**indian ocean islands**" OR "indian ocean" OR **mali** OR **malta** OR **micronesia** OR "federated states of micronesia" OR kiribati OR "marshall islands" OR nauru OR "northern mariana islands" OR **palau** OR tuvalu OR **mauritania** OR **mauritius** OR **mexico** OR **moldova** OR moldovian OR **mongolia** OR **montenegro** OR **morocco** OR ifni OR **mozambique** OR "portuguese east africa" OR **myanmar** OR burma OR **namibia** OR **nepal** OR "**netherlands antilles**" OR **nicaragua** OR **niger** OR **nigeria** OR **oman** OR muscat OR **pakistan** OR **panama** OR "**papua new guinea**" OR "new guinea" OR **paraguay** OR **peru** OR **philippines** OR philipines OR phillipines OR phillippines OR **poland** OR "polish people's republic" OR **portugal** OR portuguese republic OR "**puerto rico**" OR **romania** OR **russia** OR "russian federation" OR ussr OR "soviet union" OR "union of soviet socialist republics" OR **rwanda** OR ruanda OR **samoa** OR "pacific islands" OR polynesia OR "samoan islands" OR "navigator island" OR "navigator islands" OR **"sao tome and principe"** OR "**saudi arabia**" OR **senegal** OR **serbia** OR **seychelles** OR "**sierra leone**" OR **slovakia** OR "slovak republic" OR **slovenia** OR **melanesia** OR "solomon island" OR "solomon islands" OR "norfolk island" OR "norfolk islands" OR **somalia** OR "**south africa**" OR "**south** **sudan**" OR "**sri lanka**" OR ceylon OR **"saint kitts and nevis"** OR "st. kitts and nevis" OR "**saint lucia**" OR "st. lucia" OR **"saint vincent and the grenadines"** OR "saint vincent" OR "st. vincent" OR grenadines OR **sudan** OR **suriname** OR surinam OR "dutch guiana" OR "netherlands guiana" OR **syria** OR "syrian arab republic" OR **tajikistan** OR tadjikistan OR tadzhikistan OR tadzhik OR **tanzania** OR tanganyika OR **thailand** OR siam OR "**timor leste**" OR "east timor" OR **togo** OR "togolese republic" OR **tonga** OR **"trinidad and tobago"** OR trinidad OR tobago OR **tunisia** OR **turkey** OR **turkmenistan** OR turkmen OR **uganda** OR **ukraine** OR **uruguay** OR **uzbekistan** OR uzbek OR **vanuatu** OR "new hebrides" OR **venezuela** OR **vietnam** OR "viet nam" OR "**middle east**" OR "west bank" OR gaza OR palestine OR **yemen** OR **yugoslavia** OR **zambia** OR **zimbabwe** OR "northern rhodesia" OR "global south" OR "**africa south of the sahara**" OR "sub-saharan africa" OR "subsaharan africa" OR "**africa, central**" OR "central africa" OR "**africa, northern**" OR "north africa" OR "northern africa" OR magreb OR maghrib OR sahara OR "**africa, southern**" OR "southern africa" OR "**africa, eastern**" OR "east africa" OR "eastern africa" OR "**africa, western**" OR "west africa" OR "western africa" OR "**west indies**" OR "**indian ocean islands**" OR **caribbean** OR "**central america**" OR "**latin america**" OR "south and central america" OR "**south america**" OR "**asia, central**" OR "central asia" OR "**asia, northern**" OR "north asia" OR "northern asia" OR "**asia, southeastern**" OR "southeastern asia" OR "south eastern asia" OR "southeast asia" OR "south east asia" OR "**asia, western**" OR "western asia" OR "**europe, eastern**" OR "east europe" OR "eastern europe" OR "developing country" OR "**developing countries**" OR "developing nation#" OR "developing population#" OR "developing world" OR "less developed countr*" OR "less developed nation#" OR "less developed population#" OR "less developed world" OR "lesser developed countr*" OR "lesser developed nation#" OR "lesser developed population#" OR "lesser developed world" OR "under developed countr*" OR "under developed nation#" OR "under developed population#" OR "under developed world" OR "underdeveloped countr*" OR "underdeveloped nation#" OR "underdeveloped population#" OR "underdeveloped world" OR "middle income countr*" OR "middle income nation#" OR "middle income population#" OR "low income countr*" OR "low income nation#" OR "low income population#" OR "lower income countr*" OR "lower income nation#" OR "lower income population#" OR "underserved countr*" OR "underserved nation#" OR "underserved population#" OR "underserved world" OR "under served countr*" OR "under served nation#" OR "under served population#" OR "under served world" OR "deprived countr*" OR "deprived nation#" OR "deprived population#" OR "deprived world" OR "poor countr*" OR "poor nation#" OR "poor population#" OR "poor world" OR "poorer countr*" OR "poorer nation#" OR "poorer population#" OR "poorer world" OR "developing econom*" OR "less developed econom*" OR "lesser developed econom*" OR "under developed econom*" OR "underdeveloped econom*" OR "middle income econom*" OR "low income econom*" OR "lower income econom*" OR "low gdp" OR "low gnp" OR "low gross domestic" OR "low gross national" OR "lower gdp" OR "lower gnp" OR "lower gross domestic" OR "lower gross national" OR lmic OR lmics OR "third world" OR "lami countr*" OR "transitional countr*" OR "emerging economies" OR "emerging nation#") | 292083 |
| 29 | MH(**afghanistan** OR **albania** OR **algeria** OR "**american samoa**" OR **angola** OR **"antigua and barbuda"** OR antigua OR barbuda OR **argentina** OR **armenia** OR armenian OR **aruba** OR **azerbaijan** OR **bahrain** OR **bangladesh** OR **barbados** OR "**republic of belarus**" OR belarus OR byelarus OR belorussia OR byelorussian OR **belize** OR "british honduras" OR **benin** OR dahomey OR **bhutan** OR **bolivia** OR **"bosnia and herzegovina"** OR Bosnia-Herzegovina OR bosnia OR herzegovina OR **botswana** OR bechuanaland OR **brazil** OR brasil OR **bulgaria** OR "**burkina faso**" OR "burkina fasso" OR "upper volta" OR **burundi** OR urundi OR "**cabo verde**" OR "cape verde" OR **cambodia** OR kampuchea OR "khmer republic" OR **cameroon** OR cameron OR cameroun OR "**central african republic**" OR "ubangi shari" OR **chad** OR **chile** OR **china** OR **colombia** OR **comoros** OR "comoro islands" OR "iles comores" OR mayotte OR "**democratic republic of the congo**" OR "democratic republic congo" OR **congo** OR zaire OR "**costa rica**" OR **"cote d’ivoire"** OR "cote d’ ivoire" OR "cote divoire" OR "cote d ivoire" OR "ivory coast" OR **croatia** OR **cuba** OR **cyprus** OR "**czech republic**" OR czechoslovakia OR **djibouti** OR "french somaliland" OR **dominica** OR "**dominican republic**" OR **ecuador** OR **egypt** OR "united arab republic" OR "**el salvador**" OR "**equatorial guinea**" OR "spanish guinea" OR **eritrea** OR **estonia** OR eswatini OR **swaziland** OR **ethiopia** OR **fiji** OR **gabon** OR "gabonese republic" OR **gambia** OR **"georgia (republic)"** OR georgian OR **ghana** OR "gold coast" OR **gibraltar** OR **greece** OR **grenada** OR **guam** OR **guatemala** OR **guinea** OR "**guinea bissau**" OR **guyana** OR "british guiana" OR **haiti** OR hispaniola OR **honduras** OR **hungary** OR **india** OR **indonesia** OR timor OR **iran** OR **iraq** OR "isle of man" OR **jamaica** OR **jordan** OR **kazakhstan** OR kazakh OR **kenya** OR "**democratic people’s republic of korea"** OR "**republic of korea**" OR "north korea" OR "south korea" OR korea OR **kosovo** OR **kyrgyzstan** OR kirghizia OR kirgizstan OR "kyrgyz republic" OR kirghiz OR **laos** OR "lao pdr" OR "lao people's democratic republic" OR **latvia** OR **lebanon** OR "lebanese republic" OR **lesotho** OR basutoland OR **liberia** OR **libya** OR "libyan arab jamahiriya" OR **lithuania** OR **macau** OR macao OR "**republic of north macedonia**" OR macedonia OR **madagascar** OR "malagasy republic" OR **malawi** OR nyasaland OR **malaysia** OR "malay federation" OR malaya federation OR maldives OR "**indian ocean islands**" OR "indian ocean" OR **mali** OR **malta** OR **micronesia** OR "federated states of micronesia" OR kiribati OR "marshall islands" OR nauru OR "northern mariana islands" OR **palau** OR tuvalu OR **mauritania** OR **mauritius** OR **mexico** OR **moldova** OR moldovian OR **mongolia** OR **montenegro** OR **morocco** OR ifni OR **mozambique** OR "portuguese east africa" OR **myanmar** OR burma OR **namibia** OR **nepal** OR "**netherlands antilles**" OR **nicaragua** OR **niger** OR **nigeria** OR **oman** OR muscat OR **pakistan** OR **panama** OR "**papua new guinea**" OR "new guinea" OR **paraguay** OR **peru** OR **philippines** OR philipines OR phillipines OR phillippines OR **poland** OR "polish people's republic" OR **portugal** OR portuguese republic OR "**puerto rico**" OR **romania** OR **russia** OR "russian federation" OR ussr OR "soviet union" OR "union of soviet socialist republics" OR **rwanda** OR ruanda OR **samoa** OR "pacific islands" OR polynesia OR "samoan islands" OR "navigator island" OR "navigator islands" OR **"sao tome and principe"** OR "**saudi arabia**" OR **senegal** OR **serbia** OR **seychelles** OR "**sierra leone**" OR **slovakia** OR "slovak republic" OR **slovenia** OR **melanesia** OR "solomon island" OR "solomon islands" OR "norfolk island" OR "norfolk islands" OR **somalia** OR "**south africa**" OR "**south** **sudan**" OR "**sri lanka**" OR ceylon OR **"saint kitts and nevis"** OR "st. kitts and nevis" OR "**saint lucia**" OR "st. lucia" OR **"saint vincent and the grenadines"** OR "saint vincent" OR "st. vincent" OR grenadines OR **sudan** OR **suriname** OR surinam OR "dutch guiana" OR "netherlands guiana" OR **syria** OR "syrian arab republic" OR **tajikistan** OR tadjikistan OR tadzhikistan OR tadzhik OR **tanzania** OR tanganyika OR **thailand** OR siam OR "**timor leste**" OR "east timor" OR **togo** OR "togolese republic" OR **tonga** OR **"trinidad and tobago"** OR trinidad OR tobago OR **tunisia** OR **turkey** OR **turkmenistan** OR turkmen OR **uganda** OR **ukraine** OR **uruguay** OR **uzbekistan** OR uzbek OR **vanuatu** OR "new hebrides" OR **venezuela** OR **vietnam** OR "viet nam" OR "**middle east**" OR "west bank" OR gaza OR palestine OR **yemen** OR **yugoslavia** OR **zambia** OR **zimbabwe** OR "northern rhodesia" OR "global south" OR "**africa south of the sahara**" OR "sub-saharan africa" OR "subsaharan africa" OR "**africa, central**" OR "central africa" OR "**africa, northern**" OR "north africa" OR "northern africa" OR magreb OR maghrib OR sahara OR "**africa, southern**" OR "southern africa" OR "**africa, eastern**" OR "east africa" OR "eastern africa" OR "**africa, western**" OR "west africa" OR "western africa" OR "**west indies**" OR "**indian ocean islands**" OR **caribbean** OR "**central america**" OR "**latin america**" OR "south and central america" OR "**south america**" OR "**asia, central**" OR "central asia" OR "**asia, northern**" OR "north asia" OR "northern asia" OR "**asia, southeastern**" OR "southeastern asia" OR "south eastern asia" OR "southeast asia" OR "south east asia" OR "**asia, western**" OR "western asia" OR "**europe, eastern**" OR "east europe" OR "eastern europe" OR "developing country" OR "**developing countries**" OR "developing nation#" OR "developing population#" OR "developing world" OR "less developed countr*" OR "less developed nation#" OR "less developed population#" OR "less developed world" OR "lesser developed countr*" OR "lesser developed nation#" OR "lesser developed population#" OR "lesser developed world" OR "under developed countr*" OR "under developed nation#" OR "under developed population#" OR "under developed world" OR "underdeveloped countr*" OR "underdeveloped nation#" OR "underdeveloped population#" OR "underdeveloped world" OR "middle income countr*" OR "middle income nation#" OR "middle income population#" OR "low income countr*" OR "low income nation#" OR "low income population#" OR "lower income countr*" OR "lower income nation#" OR "lower income population#" OR "underserved countr*" OR "underserved nation#" OR "underserved population#" OR "underserved world" OR "under served countr*" OR "under served nation#" OR "under served population#" OR "under served world" OR "deprived countr*" OR "deprived nation#" OR "deprived population#" OR "deprived world" OR "poor countr*" OR "poor nation#" OR "poor population#" OR "poor world" OR "poorer countr*" OR "poorer nation#" OR "poorer population#" OR "poorer world" OR "developing econom*" OR "less developed econom*" OR "lesser developed econom*" OR "under developed econom*" OR "underdeveloped econom*" OR "middle income econom*" OR "low income econom*" OR "lower income econom*" OR "low gdp" OR "low gnp" OR "low gross domestic" OR "low gross national" OR "lower gdp" OR "lower gnp" OR "lower gross domestic" OR "lower gross national" OR lmic OR lmics OR "third world" OR "lami countr*" OR "transitional countr*" OR "emerging economies" OR "emerging nation#") | 483989 |
| 30 | S27 OR S28 OR S29 | 601662 |
| 31 | S4 AND S26 AND S30 | 503 |
|  |  |  |

| Database | Global Health |  |
| --- | --- | --- |
| Platform | Ebsco |  |
| Date Late Searched | September 6, 2022 |  |
| Number of Results | 1422 |  |
| Notes |  |  |
| 1 | TI(Sepsis OR septicemia# OR "blood poisoning#" OR pyemia# OR pyohemia# OR "bloodstream infection#") | 14354 |
| 2 | AB(Sepsis OR septicemia# OR "blood poisoning#" OR pyemia# OR pyohemia# OR "bloodstream infection#") | 32552 |
| 3 | DE "sepsis" | 19908 |
| 4 | S1 OR S2 OR S3 | 37463 |
| 5 | TI((sepsis OR septic) W3 (Prognos* OR algorithm OR tool# OR score# OR predictor# OR prediction OR calculator# OR index* OR diagnos* OR instrument# OR model# OR mortality OR "severity score" OR "mortality prediction" OR "risk stratification")) | 481 |
| 6 | AB((sepsis OR septic) W3 (Prognos* OR algorithm OR tool# OR score# OR predictor# OR prediction OR calculator# OR index* OR diagnos* OR instrument# OR model# OR mortality OR "severity score" OR "mortality prediction" OR "risk stratification")) | 2746 |
| 7 | TI((p#ediatric OR modified OR national) W1 ("early warning score")) | 30 |
| 7 | AB((p#ediatric OR modified OR national) W1 ("early warning score")) | 152 |
| 9 | TI("PEWS" OR "EWS" OR "MEWS" OR "NEWS") | 731 |
| 10 | AB("PEWS" OR "EWS" OR "MEWS" OR "NEWS") | 3232 |
| 11 | TI("sequential organ failure assessment" OR "sofa" OR "qsofa" OR "pSOFA") | 200 |
| 12 | AB("sequential organ failure assessment" OR "sofa" OR "qsofa" OR "pSOFA") | 2284 |
| 13 | TI("systemic inflammatory response syndrome" OR "sirs") | 303 |
| 14 | AB("systemic inflammatory response syndrome" OR "sirs") | 2188 |
| 15 | TI("universal vital assessment" OR "UVA") | 281 |
| 16 | AB("universal vital assessment" OR "UVA") | 1076 |
| 17 | TI("mortality in severe sepsis in the ED" OR "mortality in severe sepsis in the emergency department" OR "MISSED" OR "sMISSED") | 1002 |
| 18 | AB("mortality in severe sepsis in the ED" OR "mortality in severe sepsis in the emergency department" OR "MISSED" OR "sMISSED") | 8699 |
| 19 | TI("risk-stratification of ED suspected sepsis" OR "risk-stratification of emergency department suspected sepsis" OR "REDS score") | 2 |
| 20 | AB("risk-stratification of ED suspected sepsis" OR "risk-stratification of emergency department suspected sepsis" OR "REDS score") | 2 |
| 21 | TI("p#ediatric logistic organ dysfunction 2 score" OR PELOD-2 OR PELOD) | 5 |
| 22 | AB("p#ediatric logistic organ dysfunction 2 score" OR PELOD-2 OR PELOD) | 32 |
| 23 | TI("neosep severity and recovery score*" OR "neosep severity score*" OR "neosep recovery score*") | 0 |
| 24 | AB("neosep severity and recovery score*" OR "neosep severity score*" OR "neosep recovery score*") | 0 |
| 25 | S5 OR S6 OR S7 OR S8 OR S9 OR S10 OR S11 OR S12 OR S13 OR S14 OR S15 OR S16 OR S17 OR S18 OR S19 OR S20 OR S21 OR S22 OR S23 OR S24 | 20550 |
| 26 | TI(**afghanistan** OR **albania** OR **algeria** OR "**american samoa**" OR **angola** OR **"antigua and barbuda"** OR antigua OR barbuda OR **argentina** OR **armenia** OR armenian OR **aruba** OR **azerbaijan** OR **bahrain** OR **bangladesh** OR **barbados** OR "**republic of belarus**" OR belarus OR byelarus OR belorussia OR byelorussian OR **belize** OR "british honduras" OR **benin** OR dahomey OR **bhutan** OR **bolivia** OR **"bosnia and herzegovina"** OR Bosnia-Herzegovina OR bosnia OR herzegovina OR **botswana** OR bechuanaland OR **brazil** OR brasil OR **bulgaria** OR "**burkina faso**" OR "burkina fasso" OR "upper volta" OR **burundi** OR urundi OR "**cabo verde**" OR "cape verde" OR **cambodia** OR kampuchea OR "khmer republic" OR **cameroon** OR cameron OR cameroun OR "**central african republic**" OR "ubangi shari" OR **chad** OR **chile** OR **china** OR **colombia** OR **comoros** OR "comoro islands" OR "iles comores" OR mayotte OR "**democratic republic of the congo**" OR "democratic republic congo" OR **congo** OR zaire OR "**costa rica**" OR **"cote d’ivoire"** OR "cote d’ ivoire" OR "cote divoire" OR "cote d ivoire" OR "ivory coast" OR **croatia** OR **cuba** OR **cyprus** OR "**czech republic**" OR czechoslovakia OR **djibouti** OR "french somaliland" OR **dominica** OR "**dominican republic**" OR **ecuador** OR **egypt** OR "united arab republic" OR "**el salvador**" OR "**equatorial guinea**" OR "spanish guinea" OR **eritrea** OR **estonia** OR eswatini OR **swaziland** OR **ethiopia** OR **fiji** OR **gabon** OR "gabonese republic" OR **gambia** OR **"georgia (republic)"** OR georgian OR **ghana** OR "gold coast" OR **gibraltar** OR **greece** OR **grenada** OR **guam** OR **guatemala** OR **guinea** OR "**guinea bissau**" OR **guyana** OR "british guiana" OR **haiti** OR hispaniola OR **honduras** OR **hungary** OR **india** OR **indonesia** OR timor OR **iran** OR **iraq** OR "isle of man" OR **jamaica** OR **jordan** OR **kazakhstan** OR kazakh OR **kenya** OR "**democratic people’s republic of korea"** OR "**republic of korea**" OR "north korea" OR "south korea" OR korea OR **kosovo** OR **kyrgyzstan** OR kirghizia OR kirgizstan OR "kyrgyz republic" OR kirghiz OR **laos** OR "lao pdr" OR "lao people's democratic republic" OR **latvia** OR **lebanon** OR "lebanese republic" OR **lesotho** OR basutoland OR **liberia** OR **libya** OR "libyan arab jamahiriya" OR **lithuania** OR **macau** OR macao OR "**republic of north macedonia**" OR macedonia OR **madagascar** OR "malagasy republic" OR **malawi** OR nyasaland OR **malaysia** OR "malay federation" OR malaya federation OR maldives OR "**indian ocean islands**" OR "indian ocean" OR **mali** OR **malta** OR **micronesia** OR "federated states of micronesia" OR kiribati OR "marshall islands" OR nauru OR "northern mariana islands" OR **palau** OR tuvalu OR **mauritania** OR **mauritius** OR **mexico** OR **moldova** OR moldovian OR **mongolia** OR **montenegro** OR **morocco** OR ifni OR **mozambique** OR "portuguese east africa" OR **myanmar** OR burma OR **namibia** OR **nepal** OR "**netherlands antilles**" OR **nicaragua** OR **niger** OR **nigeria** OR **oman** OR muscat OR **pakistan** OR **panama** OR "**papua new guinea**" OR "new guinea" OR **paraguay** OR **peru** OR **philippines** OR philipines OR phillipines OR phillippines OR **poland** OR "polish people's republic" OR **portugal** OR portuguese republic OR "**puerto rico**" OR **romania** OR **russia** OR "russian federation" OR ussr OR "soviet union" OR "union of soviet socialist republics" OR **rwanda** OR ruanda OR **samoa** OR "pacific islands" OR polynesia OR "samoan islands" OR "navigator island" OR "navigator islands" OR **"sao tome and principe"** OR "**saudi arabia**" OR **senegal** OR **serbia** OR **seychelles** OR "**sierra leone**" OR **slovakia** OR "slovak republic" OR **slovenia** OR **melanesia** OR "solomon island" OR "solomon islands" OR "norfolk island" OR "norfolk islands" OR **somalia** OR "**south africa**" OR "**south** **sudan**" OR "**sri lanka**" OR ceylon OR **"saint kitts and nevis"** OR "st. kitts and nevis" OR "**saint lucia**" OR "st. lucia" OR **"saint vincent and the grenadines"** OR "saint vincent" OR "st. vincent" OR grenadines OR **sudan** OR **suriname** OR surinam OR "dutch guiana" OR "netherlands guiana" OR **syria** OR "syrian arab republic" OR **tajikistan** OR tadjikistan OR tadzhikistan OR tadzhik OR **tanzania** OR tanganyika OR **thailand** OR siam OR "**timor leste**" OR "east timor" OR **togo** OR "togolese republic" OR **tonga** OR **"trinidad and tobago"** OR trinidad OR tobago OR **tunisia** OR **turkey** OR **turkmenistan** OR turkmen OR **uganda** OR **ukraine** OR **uruguay** OR **uzbekistan** OR uzbek OR **vanuatu** OR "new hebrides" OR **venezuela** OR **vietnam** OR "viet nam" OR "**middle east**" OR "west bank" OR gaza OR palestine OR **yemen** OR **yugoslavia** OR **zambia** OR **zimbabwe** OR "northern rhodesia" OR "global south" OR "**africa south of the sahara**" OR "sub-saharan africa" OR "subsaharan africa" OR "**africa, central**" OR "central africa" OR "**africa, northern**" OR "north africa" OR "northern africa" OR magreb OR maghrib OR sahara OR "**africa, southern**" OR "southern africa" OR "**africa, eastern**" OR "east africa" OR "eastern africa" OR "**africa, western**" OR "west africa" OR "western africa" OR "**west indies**" OR "**indian ocean islands**" OR **caribbean** OR "**central america**" OR "**latin america**" OR "south and central america" OR "**south america**" OR "**asia, central**" OR "central asia" OR "**asia, northern**" OR "north asia" OR "northern asia" OR "**asia, southeastern**" OR "southeastern asia" OR "south eastern asia" OR "southeast asia" OR "south east asia" OR "**asia, western**" OR "western asia" OR "**europe, eastern**" OR "east europe" OR "eastern europe" OR "developing country" OR "**developing countries**" OR "developing nation#" OR "developing population#" OR "developing world" OR "less developed countr*" OR "less developed nation#" OR "less developed population#" OR "less developed world" OR "lesser developed countr*" OR "lesser developed nation#" OR "lesser developed population#" OR "lesser developed world" OR "under developed countr*" OR "under developed nation#" OR "under developed population#" OR "under developed world" OR "underdeveloped countr*" OR "underdeveloped nation#" OR "underdeveloped population#" OR "underdeveloped world" OR "middle income countr*" OR "middle income nation#" OR "middle income population#" OR "low income countr*" OR "low income nation#" OR "low income population#" OR "lower income countr*" OR "lower income nation#" OR "lower income population#" OR "underserved countr*" OR "underserved nation#" OR "underserved population#" OR "underserved world" OR "under served countr*" OR "under served nation#" OR "under served population#" OR "under served world" OR "deprived countr*" OR "deprived nation#" OR "deprived population#" OR "deprived world" OR "poor countr*" OR "poor nation#" OR "poor population#" OR "poor world" OR "poorer countr*" OR "poorer nation#" OR "poorer population#" OR "poorer world" OR "developing econom*" OR "less developed econom*" OR "lesser developed econom*" OR "under developed econom*" OR "underdeveloped econom*" OR "middle income econom*" OR "low income econom*" OR "lower income econom*" OR "low gdp" OR "low gnp" OR "low gross domestic" OR "low gross national" OR "lower gdp" OR "lower gnp" OR "lower gross domestic" OR "lower gross national" OR lmic OR lmics OR "third world" OR "lami countr*" OR "transitional countr*" OR "emerging economies" OR "emerging nation#") | 450191 |
| 27 | AB(**afghanistan** OR **albania** OR **algeria** OR "**american samoa**" OR **angola** OR **"antigua and barbuda"** OR antigua OR barbuda OR **argentina** OR **armenia** OR armenian OR **aruba** OR **azerbaijan** OR **bahrain** OR **bangladesh** OR **barbados** OR "**republic of belarus**" OR belarus OR byelarus OR belorussia OR byelorussian OR **belize** OR "british honduras" OR **benin** OR dahomey OR **bhutan** OR **bolivia** OR **"bosnia and herzegovina"** OR Bosnia-Herzegovina OR bosnia OR herzegovina OR **botswana** OR bechuanaland OR **brazil** OR brasil OR **bulgaria** OR "**burkina faso**" OR "burkina fasso" OR "upper volta" OR **burundi** OR urundi OR "**cabo verde**" OR "cape verde" OR **cambodia** OR kampuchea OR "khmer republic" OR **cameroon** OR cameron OR cameroun OR "**central african republic**" OR "ubangi shari" OR **chad** OR **chile** OR **china** OR **colombia** OR **comoros** OR "comoro islands" OR "iles comores" OR mayotte OR "**democratic republic of the congo**" OR "democratic republic congo" OR **congo** OR zaire OR "**costa rica**" OR **"cote d’ivoire"** OR "cote d’ ivoire" OR "cote divoire" OR "cote d ivoire" OR "ivory coast" OR **croatia** OR **cuba** OR **cyprus** OR "**czech republic**" OR czechoslovakia OR **djibouti** OR "french somaliland" OR **dominica** OR "**dominican republic**" OR **ecuador** OR **egypt** OR "united arab republic" OR "**el salvador**" OR "**equatorial guinea**" OR "spanish guinea" OR **eritrea** OR **estonia** OR eswatini OR **swaziland** OR **ethiopia** OR **fiji** OR **gabon** OR "gabonese republic" OR **gambia** OR **"georgia (republic)"** OR georgian OR **ghana** OR "gold coast" OR **gibraltar** OR **greece** OR **grenada** OR **guam** OR **guatemala** OR **guinea** OR "**guinea bissau**" OR **guyana** OR "british guiana" OR **haiti** OR hispaniola OR **honduras** OR **hungary** OR **india** OR **indonesia** OR timor OR **iran** OR **iraq** OR "isle of man" OR **jamaica** OR **jordan** OR **kazakhstan** OR kazakh OR **kenya** OR "**democratic people’s republic of korea"** OR "**republic of korea**" OR "north korea" OR "south korea" OR korea OR **kosovo** OR **kyrgyzstan** OR kirghizia OR kirgizstan OR "kyrgyz republic" OR kirghiz OR **laos** OR "lao pdr" OR "lao people's democratic republic" OR **latvia** OR **lebanon** OR "lebanese republic" OR **lesotho** OR basutoland OR **liberia** OR **libya** OR "libyan arab jamahiriya" OR **lithuania** OR **macau** OR macao OR "**republic of north macedonia**" OR macedonia OR **madagascar** OR "malagasy republic" OR **malawi** OR nyasaland OR **malaysia** OR "malay federation" OR malaya federation OR maldives OR "**indian ocean islands**" OR "indian ocean" OR **mali** OR **malta** OR **micronesia** OR "federated states of micronesia" OR kiribati OR "marshall islands" OR nauru OR "northern mariana islands" OR **palau** OR tuvalu OR **mauritania** OR **mauritius** OR **mexico** OR **moldova** OR moldovian OR **mongolia** OR **montenegro** OR **morocco** OR ifni OR **mozambique** OR "portuguese east africa" OR **myanmar** OR burma OR **namibia** OR **nepal** OR "**netherlands antilles**" OR **nicaragua** OR **niger** OR **nigeria** OR **oman** OR muscat OR **pakistan** OR **panama** OR "**papua new guinea**" OR "new guinea" OR **paraguay** OR **peru** OR **philippines** OR philipines OR phillipines OR phillippines OR **poland** OR "polish people's republic" OR **portugal** OR portuguese republic OR "**puerto rico**" OR **romania** OR **russia** OR "russian federation" OR ussr OR "soviet union" OR "union of soviet socialist republics" OR **rwanda** OR ruanda OR **samoa** OR "pacific islands" OR polynesia OR "samoan islands" OR "navigator island" OR "navigator islands" OR **"sao tome and principe"** OR "**saudi arabia**" OR **senegal** OR **serbia** OR **seychelles** OR "**sierra leone**" OR **slovakia** OR "slovak republic" OR **slovenia** OR **melanesia** OR "solomon island" OR "solomon islands" OR "norfolk island" OR "norfolk islands" OR **somalia** OR "**south africa**" OR "**south** **sudan**" OR "**sri lanka**" OR ceylon OR **"saint kitts and nevis"** OR "st. kitts and nevis" OR "**saint lucia**" OR "st. lucia" OR **"saint vincent and the grenadines"** OR "saint vincent" OR "st. vincent" OR grenadines OR **sudan** OR **suriname** OR surinam OR "dutch guiana" OR "netherlands guiana" OR **syria** OR "syrian arab republic" OR **tajikistan** OR tadjikistan OR tadzhikistan OR tadzhik OR **tanzania** OR tanganyika OR **thailand** OR siam OR "**timor leste**" OR "east timor" OR **togo** OR "togolese republic" OR **tonga** OR **"trinidad and tobago"** OR trinidad OR tobago OR **tunisia** OR **turkey** OR **turkmenistan** OR turkmen OR **uganda** OR **ukraine** OR **uruguay** OR **uzbekistan** OR uzbek OR **vanuatu** OR "new hebrides" OR **venezuela** OR **vietnam** OR "viet nam" OR "**middle east**" OR "west bank" OR gaza OR palestine OR **yemen** OR **yugoslavia** OR **zambia** OR **zimbabwe** OR "northern rhodesia" OR "global south" OR "**africa south of the sahara**" OR "sub-saharan africa" OR "subsaharan africa" OR "**africa, central**" OR "central africa" OR "**africa, northern**" OR "north africa" OR "northern africa" OR magreb OR maghrib OR sahara OR "**africa, southern**" OR "southern africa" OR "**africa, eastern**" OR "east africa" OR "eastern africa" OR "**africa, western**" OR "west africa" OR "western africa" OR "**west indies**" OR "**indian ocean islands**" OR **caribbean** OR "**central america**" OR "**latin america**" OR "south and central america" OR "**south america**" OR "**asia, central**" OR "central asia" OR "**asia, northern**" OR "north asia" OR "northern asia" OR "**asia, southeastern**" OR "southeastern asia" OR "south eastern asia" OR "southeast asia" OR "south east asia" OR "**asia, western**" OR "western asia" OR "**europe, eastern**" OR "east europe" OR "eastern europe" OR "developing country" OR "**developing countries**" OR "developing nation#" OR "developing population#" OR "developing world" OR "less developed countr*" OR "less developed nation#" OR "less developed population#" OR "less developed world" OR "lesser developed countr*" OR "lesser developed nation#" OR "lesser developed population#" OR "lesser developed world" OR "under developed countr*" OR "under developed nation#" OR "under developed population#" OR "under developed world" OR "underdeveloped countr*" OR "underdeveloped nation#" OR "underdeveloped population#" OR "underdeveloped world" OR "middle income countr*" OR "middle income nation#" OR "middle income population#" OR "low income countr*" OR "low income nation#" OR "low income population#" OR "lower income countr*" OR "lower income nation#" OR "lower income population#" OR "underserved countr*" OR "underserved nation#" OR "underserved population#" OR "underserved world" OR "under served countr*" OR "under served nation#" OR "under served population#" OR "under served world" OR "deprived countr*" OR "deprived nation#" OR "deprived population#" OR "deprived world" OR "poor countr*" OR "poor nation#" OR "poor population#" OR "poor world" OR "poorer countr*" OR "poorer nation#" OR "poorer population#" OR "poorer world" OR "developing econom*" OR "less developed econom*" OR "lesser developed econom*" OR "under developed econom*" OR "underdeveloped econom*" OR "middle income econom*" OR "low income econom*" OR "lower income econom*" OR "low gdp" OR "low gnp" OR "low gross domestic" OR "low gross national" OR "lower gdp" OR "lower gnp" OR "lower gross domestic" OR "lower gross national" OR lmic OR lmics OR "third world" OR "lami countr*" OR "transitional countr*" OR "emerging economies" OR "emerging nation#") | 791950 |
| 28 | DE(**afghanistan** OR **albania** OR **algeria** OR "**american samoa**" OR **angola** OR **"antigua and barbuda"** OR antigua OR barbuda OR **argentina** OR **armenia** OR armenian OR **aruba** OR **azerbaijan** OR **bahrain** OR **bangladesh** OR **barbados** OR "**republic of belarus**" OR belarus OR byelarus OR belorussia OR byelorussian OR **belize** OR "british honduras" OR **benin** OR dahomey OR **bhutan** OR **bolivia** OR **"bosnia and herzegovina"** OR Bosnia-Herzegovina OR bosnia OR herzegovina OR **botswana** OR bechuanaland OR **brazil** OR brasil OR **bulgaria** OR "**burkina faso**" OR "burkina fasso" OR "upper volta" OR **burundi** OR urundi OR "**cabo verde**" OR "cape verde" OR **cambodia** OR kampuchea OR "khmer republic" OR **cameroon** OR cameron OR cameroun OR "**central african republic**" OR "ubangi shari" OR **chad** OR **chile** OR **china** OR **colombia** OR **comoros** OR "comoro islands" OR "iles comores" OR mayotte OR "**democratic republic of the congo**" OR "democratic republic congo" OR **congo** OR zaire OR "**costa rica**" OR **"cote d’ivoire"** OR "cote d’ ivoire" OR "cote divoire" OR "cote d ivoire" OR "ivory coast" OR **croatia** OR **cuba** OR **cyprus** OR "**czech republic**" OR czechoslovakia OR **djibouti** OR "french somaliland" OR **dominica** OR "**dominican republic**" OR **ecuador** OR **egypt** OR "united arab republic" OR "**el salvador**" OR "**equatorial guinea**" OR "spanish guinea" OR **eritrea** OR **estonia** OR eswatini OR **swaziland** OR **ethiopia** OR **fiji** OR **gabon** OR "gabonese republic" OR **gambia** OR **"georgia (republic)"** OR georgian OR **ghana** OR "gold coast" OR **gibraltar** OR **greece** OR **grenada** OR **guam** OR **guatemala** OR **guinea** OR "**guinea bissau**" OR **guyana** OR "british guiana" OR **haiti** OR hispaniola OR **honduras** OR **hungary** OR **india** OR **indonesia** OR timor OR **iran** OR **iraq** OR "isle of man" OR **jamaica** OR **jordan** OR **kazakhstan** OR kazakh OR **kenya** OR "**democratic people’s republic of korea"** OR "**republic of korea**" OR "north korea" OR "south korea" OR korea OR **kosovo** OR **kyrgyzstan** OR kirghizia OR kirgizstan OR "kyrgyz republic" OR kirghiz OR **laos** OR "lao pdr" OR "lao people's democratic republic" OR **latvia** OR **lebanon** OR "lebanese republic" OR **lesotho** OR basutoland OR **liberia** OR **libya** OR "libyan arab jamahiriya" OR **lithuania** OR **macau** OR macao OR "**republic of north macedonia**" OR macedonia OR **madagascar** OR "malagasy republic" OR **malawi** OR nyasaland OR **malaysia** OR "malay federation" OR malaya federation OR maldives OR "**indian ocean islands**" OR "indian ocean" OR **mali** OR **malta** OR **micronesia** OR "federated states of micronesia" OR kiribati OR "marshall islands" OR nauru OR "northern mariana islands" OR **palau** OR tuvalu OR **mauritania** OR **mauritius** OR **mexico** OR **moldova** OR moldovian OR **mongolia** OR **montenegro** OR **morocco** OR ifni OR **mozambique** OR "portuguese east africa" OR **myanmar** OR burma OR **namibia** OR **nepal** OR "**netherlands antilles**" OR **nicaragua** OR **niger** OR **nigeria** OR **oman** OR muscat OR **pakistan** OR **panama** OR "**papua new guinea**" OR "new guinea" OR **paraguay** OR **peru** OR **philippines** OR philipines OR phillipines OR phillippines OR **poland** OR "polish people's republic" OR **portugal** OR portuguese republic OR "**puerto rico**" OR **romania** OR **russia** OR "russian federation" OR ussr OR "soviet union" OR "union of soviet socialist republics" OR **rwanda** OR ruanda OR **samoa** OR "pacific islands" OR polynesia OR "samoan islands" OR "navigator island" OR "navigator islands" OR **"sao tome and principe"** OR "**saudi arabia**" OR **senegal** OR **serbia** OR **seychelles** OR "**sierra leone**" OR **slovakia** OR "slovak republic" OR **slovenia** OR **melanesia** OR "solomon island" OR "solomon islands" OR "norfolk island" OR "norfolk islands" OR **somalia** OR "**south africa**" OR "**south** **sudan**" OR "**sri lanka**" OR ceylon OR **"saint kitts and nevis"** OR "st. kitts and nevis" OR "**saint lucia**" OR "st. lucia" OR **"saint vincent and the grenadines"** OR "saint vincent" OR "st. vincent" OR grenadines OR **sudan** OR **suriname** OR surinam OR "dutch guiana" OR "netherlands guiana" OR **syria** OR "syrian arab republic" OR **tajikistan** OR tadjikistan OR tadzhikistan OR tadzhik OR **tanzania** OR tanganyika OR **thailand** OR siam OR "**timor leste**" OR "east timor" OR **togo** OR "togolese republic" OR **tonga** OR **"trinidad and tobago"** OR trinidad OR tobago OR **tunisia** OR **turkey** OR **turkmenistan** OR turkmen OR **uganda** OR **ukraine** OR **uruguay** OR **uzbekistan** OR uzbek OR **vanuatu** OR "new hebrides" OR **venezuela** OR **vietnam** OR "viet nam" OR "**middle east**" OR "west bank" OR gaza OR palestine OR **yemen** OR **yugoslavia** OR **zambia** OR **zimbabwe** OR "northern rhodesia" OR "global south" OR "**africa south of the sahara**" OR "sub-saharan africa" OR "subsaharan africa" OR "**africa, central**" OR "central africa" OR "**africa, northern**" OR "north africa" OR "northern africa" OR magreb OR maghrib OR sahara OR "**africa, southern**" OR "southern africa" OR "**africa, eastern**" OR "east africa" OR "eastern africa" OR "**africa, western**" OR "west africa" OR "western africa" OR "**west indies**" OR "**indian ocean islands**" OR **caribbean** OR "**central america**" OR "**latin america**" OR "south and central america" OR "**south america**" OR "**asia, central**" OR "central asia" OR "**asia, northern**" OR "north asia" OR "northern asia" OR "**asia, southeastern**" OR "southeastern asia" OR "south eastern asia" OR "southeast asia" OR "south east asia" OR "**asia, western**" OR "western asia" OR "**europe, eastern**" OR "east europe" OR "eastern europe" OR "developing country" OR "**developing countries**" OR "developing nation#" OR "developing population#" OR "developing world" OR "less developed countr*" OR "less developed nation#" OR "less developed population#" OR "less developed world" OR "lesser developed countr*" OR "lesser developed nation#" OR "lesser developed population#" OR "lesser developed world" OR "under developed countr*" OR "under developed nation#" OR "under developed population#" OR "under developed world" OR "underdeveloped countr*" OR "underdeveloped nation#" OR "underdeveloped population#" OR "underdeveloped world" OR "middle income countr*" OR "middle income nation#" OR "middle income population#" OR "low income countr*" OR "low income nation#" OR "low income population#" OR "lower income countr*" OR "lower income nation#" OR "lower income population#" OR "underserved countr*" OR "underserved nation#" OR "underserved population#" OR "underserved world" OR "under served countr*" OR "under served nation#" OR "under served population#" OR "under served world" OR "deprived countr*" OR "deprived nation#" OR "deprived population#" OR "deprived world" OR "poor countr*" OR "poor nation#" OR "poor population#" OR "poor world" OR "poorer countr*" OR "poorer nation#" OR "poorer population#" OR "poorer world" OR "developing econom*" OR "less developed econom*" OR "lesser developed econom*" OR "under developed econom*" OR "underdeveloped econom*" OR "middle income econom*" OR "low income econom*" OR "lower income econom*" OR "low gdp" OR "low gnp" OR "low gross domestic" OR "low gross national" OR "lower gdp" OR "lower gnp" OR "lower gross domestic" OR "lower gross national" OR lmic OR lmics OR "third world" OR "lami countr*" OR "transitional countr*" OR "emerging economies" OR "emerging nation#") | 1162596 |
| 29 | S26 OR S27 OR S28 | 1280796 |
| 30 | S4 AND S25 AND S29 | 1422 |
|  |  |  |

| Database | Web of Science |  |
| --- | --- | --- |
| Platform | Clarivate |  |
| Date Late Searched | September 6, 2022 |  |
| Number of Results | 1343 |  |
| Notes |  |  |
| 1 | TI=(Sepsis OR septicemia$ OR "blood poisoning$" OR pyemia$ OR pyohemia$ OR "bloodstream infection$") | 57942 |
| 2 | AB=(Sepsis OR septicemia$ OR "blood poisoning$" OR pyemia$ OR pyohemia$ OR "bloodstream infection$") | 98997 |
| 3 | #1 OR #2 | 127801 |
| 4 | TI=((sepsis OR septic) NEAR/3 (Prognos* OR algorithm OR tool$ OR score$ OR predictor$ OR prediction OR calculator$ OR index* OR diagnos* OR instrument$ OR model$ OR mortality OR "severity score" OR "mortality prediction" OR "risk stratification")) | 7383 |
| 5 | AB=((sepsis OR septic) NEAR/3 (Prognos* OR algorithm OR tool$ OR score$ OR predictor$ OR prediction OR calculator$ OR index* OR diagnos* OR instrument$ OR model$ OR mortality OR "severity score" OR "mortality prediction" OR "risk stratification")) | 19144 |
| 6 | TI=((p$ediatric OR modified OR national) NEAR/1 ("early warning score")) | 375 |
| 7 | AB=((p$ediatric OR modified OR national) NEAR/1 ("early warning score")) | 779 |
| 8 | TI=("PEWS" OR "EWS" OR "MEWS" OR "NEWS") | 39011 |
| 9 | AB=("PEWS" OR "EWS" OR "MEWS" OR "NEWS") | 49857 |
| 10 | TI=("sequential organ failure assessment" OR "sofa" OR "qsofa" OR "pSOFA") | 1280 |
| 11 | AB=("sequential organ failure assessment" OR "sofa" OR "qsofa" OR "pSOFA") | 7224 |
| 12 | TI=("systemic inflammatory response syndrome" OR "sirs") | 2367 |
| 13 | AB=("systemic inflammatory response syndrome" OR "sirs") | 8410 |
| 14 | TI=("universal vital assessment" OR "UVA") | 3630 |
| 15 | AB=("universal vital assessment" OR "UVA") | 10173 |
| 16 | TI=("mortality in severe sepsis in the ED" OR "mortality in severe sepsis in the emergency department" OR "MISSED" OR "sMISSED") | 9648 |
| 17 | AB=("mortality in severe sepsis in the ED" OR "mortality in severe sepsis in the emergency department" OR "MISSED" OR "sMISSED") | 51042 |
| 18 | TI=("risk-stratification of ED suspected sepsis" OR "risk-stratification of emergency department suspected sepsis" OR "REDS score") | 3 |
| 19 | AB=("risk-stratification of ED suspected sepsis" OR "risk-stratification of emergency department suspected sepsis" OR "REDS score") | 2 |
| 20 | TI=("p$ediatric logistic organ dysfunction 2 score" OR PELOD-2 OR PELOD) | 39 |
| 21 | AB=("p$ediatric logistic organ dysfunction 2 score" OR PELOD-2 OR PELOD) | 158 |
| 22 | TI=("neosep severity and recovery score*" OR "neosep severity score*" OR "neosep recovery score*") | 0 |
| 23 | AB=("neosep severity and recovery score*" OR "neosep severity score*" OR "neosep recovery score*") | 0 |
| 24 | #4 OR #5 OR #6 OR #7 OR #8 OR #9 OR #10 OR #11 OR #12 OR #13 OR #14 OR #15 OR #16 OR #17 OR #18 OR #19 OR #20 OR #21 OR #22 OR #23 | 183770 |
| 25 | TI=(**afghanistan** OR **albania** OR **algeria** OR "**american samoa**" OR **angola** OR **"antigua and barbuda"** OR antigua OR barbuda OR **argentina** OR **armenia** OR armenian OR **aruba** OR **azerbaijan** OR **bahrain** OR **bangladesh** OR **barbados** OR "**republic of belarus**" OR belarus OR byelarus OR belorussia OR byelorussian OR **belize** OR "british honduras" OR **benin** OR dahomey OR **bhutan** OR **bolivia** OR **"bosnia and herzegovina"** OR Bosnia-Herzegovina OR bosnia OR herzegovina OR **botswana** OR bechuanaland OR **brazil** OR brasil OR **bulgaria** OR "**burkina faso**" OR "burkina fasso" OR "upper volta" OR **burundi** OR urundi OR "**cabo verde**" OR "cape verde" OR **cambodia** OR kampuchea OR "khmer republic" OR **cameroon** OR cameron OR cameroun OR "**central african republic**" OR "ubangi shari" OR **chad** OR **chile** OR **china** OR **colombia** OR **comoros** OR "comoro islands" OR "iles comores" OR mayotte OR "**democratic republic of the congo**" OR "democratic republic congo" OR **congo** OR zaire OR "**costa rica**" OR **"cote d’ivoire"** OR "cote d’ ivoire" OR "cote divoire" OR "cote d ivoire" OR "ivory coast" OR **croatia** OR **cuba** OR **cyprus** OR "**czech republic**" OR czechoslovakia OR **djibouti** OR "french somaliland" OR **dominica** OR "**dominican republic**" OR **ecuador** OR **egypt** OR "united arab republic" OR "**el salvador**" OR "**equatorial guinea**" OR "spanish guinea" OR **eritrea** OR **estonia** OR eswatini OR **swaziland** OR **ethiopia** OR **fiji** OR **gabon** OR "gabonese republic" OR **gambia** OR **"georgia (republic)"** OR georgian OR **ghana** OR "gold coast" OR **gibraltar** OR **greece** OR **grenada** OR **guam** OR **guatemala** OR **guinea** OR "**guinea bissau**" OR **guyana** OR "british guiana" OR **haiti** OR hispaniola OR **honduras** OR **hungary** OR **india** OR **indonesia** OR timor OR **iran** OR **iraq** OR "isle of man" OR **jamaica** OR **jordan** OR **kazakhstan** OR kazakh OR **kenya** OR "**democratic people’s republic of korea"** OR "**republic of korea**" OR "north korea" OR "south korea" OR korea OR **kosovo** OR **kyrgyzstan** OR kirghizia OR kirgizstan OR "kyrgyz republic" OR kirghiz OR **laos** OR "lao pdr" OR "lao people's democratic republic" OR **latvia** OR **lebanon** OR "lebanese republic" OR **lesotho** OR basutoland OR **liberia** OR **libya** OR "libyan arab jamahiriya" OR **lithuania** OR **macau** OR macao OR "**republic of north macedonia**" OR macedonia OR **madagascar** OR "malagasy republic" OR **malawi** OR nyasaland OR **malaysia** OR "malay federation" OR "malaya federation" OR maldives OR "**indian ocean islands**" OR "indian ocean" OR **mali** OR **malta** OR **micronesia** OR "federated states of micronesia" OR kiribati OR "marshall islands" OR nauru OR "northern mariana islands" OR **palau** OR tuvalu OR **mauritania** OR **mauritius** OR **mexico** OR **moldova** OR moldovian OR **mongolia** OR **montenegro** OR **morocco** OR ifni OR **mozambique** OR "portuguese east africa" OR **myanmar** OR burma OR **namibia** OR **nepal** OR "**netherlands antilles**" OR **nicaragua** OR **niger** OR **nigeria** OR **oman** OR muscat OR **pakistan** OR **panama** OR "**papua new guinea**" OR "new guinea" OR **paraguay** OR **peru** OR **philippines** OR philipines OR phillipines OR phillippines OR **poland** OR "polish people's republic" OR **portugal** OR portuguese republic OR "**puerto rico**" OR **romania** OR **russia** OR "russian federation" OR ussr OR "soviet union" OR "union of soviet socialist republics" OR **rwanda** OR ruanda OR **samoa** OR "pacific islands" OR polynesia OR "samoan islands" OR "navigator island" OR "navigator islands" OR **"sao tome and principe"** OR "**saudi arabia**" OR **senegal** OR **serbia** OR **seychelles** OR "**sierra leone**" OR **slovakia** OR "slovak republic" OR **slovenia** OR **melanesia** OR "solomon island" OR "solomon islands" OR "norfolk island" OR "norfolk islands" OR **somalia** OR "**south africa**" OR "**south** **sudan**" OR "**sri lanka**" OR ceylon OR **"saint kitts and nevis"** OR "st. kitts and nevis" OR "**saint lucia**" OR "st. lucia" OR **"saint vincent and the grenadines"** OR "saint vincent" OR "st. vincent" OR grenadines OR **sudan** OR **suriname** OR surinam OR "dutch guiana" OR "netherlands guiana" OR **syria** OR "syrian arab republic" OR **tajikistan** OR tadjikistan OR tadzhikistan OR tadzhik OR **tanzania** OR tanganyika OR **thailand** OR siam OR "**timor leste**" OR "east timor" OR **togo** OR "togolese republic" OR **tonga** OR **"trinidad and tobago"** OR trinidad OR tobago OR **tunisia** OR **turkey** OR **turkmenistan** OR turkmen OR **uganda** OR **ukraine** OR **uruguay** OR **uzbekistan** OR uzbek OR **vanuatu** OR "new hebrides" OR **venezuela** OR **vietnam** OR "viet nam" OR "**middle east**" OR "west bank" OR gaza OR palestine OR **yemen** OR **yugoslavia** OR **zambia** OR **zimbabwe** OR "northern rhodesia" OR "global south" OR "**africa south of the sahara**" OR "sub-saharan africa" OR "subsaharan africa" OR "**africa, central**" OR "central africa" OR "**africa, northern**" OR "north africa" OR "northern africa" OR magreb OR maghrib OR sahara OR "**africa, southern**" OR "southern africa" OR "**africa, eastern**" OR "east africa" OR "eastern africa" OR "**africa, western**" OR "west africa" OR "western africa" OR "**west indies**" OR "**indian ocean islands**" OR **caribbean** OR "**central america**" OR "**latin america**" OR "south and central america" OR "**south america**" OR "**asia, central**" OR "central asia" OR "**asia, northern**" OR "north asia" OR "northern asia" OR "**asia, southeastern**" OR "southeastern asia" OR "south eastern asia" OR "southeast asia" OR "south east asia" OR "**asia, western**" OR "western asia" OR "**europe, eastern**" OR "east europe" OR "eastern europe" OR "developing country" OR "**developing countries**" OR "developing nation$" OR "developing population$" OR "developing world" OR "less developed countr*" OR "less developed nation$" OR "less developed population$" OR "less developed world" OR "lesser developed countr*" OR "lesser developed nation$" OR "lesser developed population$" OR "lesser developed world" OR "under developed countr*" OR "under developed nation$" OR "under developed population$" OR "under developed world" OR "underdeveloped countr*" OR "underdeveloped nation$" OR "underdeveloped population$" OR "underdeveloped world" OR "middle income countr*" OR "middle income nation$" OR "middle income population$" OR "low income countr*" OR "low income nation$" OR "low income population$" OR "lower income countr*" OR "lower income nation$" OR "lower income population$" OR "underserved countr*" OR "underserved nation$" OR "underserved population$" OR "underserved world" OR "under served countr*" OR "under served nation$" OR "under served population$" OR "under served world" OR "deprived countr*" OR "deprived nation$" OR "deprived population$" OR "deprived world" OR "poor countr*" OR "poor nation$" OR "poor population$" OR "poor world" OR "poorer countr*" OR "poorer nation$" OR "poorer population$" OR "poorer world" OR "developing econom*" OR "less developed econom*" OR "lesser developed econom*" OR "under developed econom*" OR "underdeveloped econom*" OR "middle income econom*" OR "low income econom*" OR "lower income econom*" OR "low gdp" OR "low gnp" OR "low gross domestic" OR "low gross national" OR "lower gdp" OR "lower gnp" OR "lower gross domestic" OR "lower gross national" OR lmic OR lmics OR "third world" OR "lami countr*" OR "transitional countr*" OR "emerging economies" OR "emerging nation$") | 2436628 |
| 26 | AB=(**afghanistan** OR **albania** OR **algeria** OR "**american samoa**" OR **angola** OR **"antigua and barbuda"** OR antigua OR barbuda OR **argentina** OR **armenia** OR armenian OR **aruba** OR **azerbaijan** OR **bahrain** OR **bangladesh** OR **barbados** OR "**republic of belarus**" OR belarus OR byelarus OR belorussia OR byelorussian OR **belize** OR "british honduras" OR **benin** OR dahomey OR **bhutan** OR **bolivia** OR **"bosnia and herzegovina"** OR Bosnia-Herzegovina OR bosnia OR herzegovina OR **botswana** OR bechuanaland OR **brazil** OR brasil OR **bulgaria** OR "**burkina faso**" OR "burkina fasso" OR "upper volta" OR **burundi** OR urundi OR "**cabo verde**" OR "cape verde" OR **cambodia** OR kampuchea OR "khmer republic" OR **cameroon** OR cameron OR cameroun OR "**central african republic**" OR "ubangi shari" OR **chad** OR **chile** OR **china** OR **colombia** OR **comoros** OR "comoro islands" OR "iles comores" OR mayotte OR "**democratic republic of the congo**" OR "democratic republic congo" OR **congo** OR zaire OR "**costa rica**" OR **"cote d’ivoire"** OR "cote d’ ivoire" OR "cote divoire" OR "cote d ivoire" OR "ivory coast" OR **croatia** OR **cuba** OR **cyprus** OR "**czech republic**" OR czechoslovakia OR **djibouti** OR "french somaliland" OR **dominica** OR "**dominican republic**" OR **ecuador** OR **egypt** OR "united arab republic" OR "**el salvador**" OR "**equatorial guinea**" OR "spanish guinea" OR **eritrea** OR **estonia** OR eswatini OR **swaziland** OR **ethiopia** OR **fiji** OR **gabon** OR "gabonese republic" OR **gambia** OR **"georgia (republic)"** OR georgian OR **ghana** OR "gold coast" OR **gibraltar** OR **greece** OR **grenada** OR **guam** OR **guatemala** OR **guinea** OR "**guinea bissau**" OR **guyana** OR "british guiana" OR **haiti** OR hispaniola OR **honduras** OR **hungary** OR **india** OR **indonesia** OR timor OR **iran** OR **iraq** OR "isle of man" OR **jamaica** OR **jordan** OR **kazakhstan** OR kazakh OR **kenya** OR "**democratic people’s republic of korea"** OR "**republic of korea**" OR "north korea" OR "south korea" OR korea OR **kosovo** OR **kyrgyzstan** OR kirghizia OR kirgizstan OR "kyrgyz republic" OR kirghiz OR **laos** OR "lao pdr" OR "lao people's democratic republic" OR **latvia** OR **lebanon** OR "lebanese republic" OR **lesotho** OR basutoland OR **liberia** OR **libya** OR "libyan arab jamahiriya" OR **lithuania** OR **macau** OR macao OR "**republic of north macedonia**" OR macedonia OR **madagascar** OR "malagasy republic" OR **malawi** OR nyasaland OR **malaysia** OR "malay federation" OR "malaya federation" OR maldives OR "**indian ocean islands**" OR "indian ocean" OR **mali** OR **malta** OR **micronesia** OR "federated states of micronesia" OR kiribati OR "marshall islands" OR nauru OR "northern mariana islands" OR **palau** OR tuvalu OR **mauritania** OR **mauritius** OR **mexico** OR **moldova** OR moldovian OR **mongolia** OR **montenegro** OR **morocco** OR ifni OR **mozambique** OR "portuguese east africa" OR **myanmar** OR burma OR **namibia** OR **nepal** OR "**netherlands antilles**" OR **nicaragua** OR **niger** OR **nigeria** OR **oman** OR muscat OR **pakistan** OR **panama** OR "**papua new guinea**" OR "new guinea" OR **paraguay** OR **peru** OR **philippines** OR philipines OR phillipines OR phillippines OR **poland** OR "polish people's republic" OR **portugal** OR portuguese republic OR "**puerto rico**" OR **romania** OR **russia** OR "russian federation" OR ussr OR "soviet union" OR "union of soviet socialist republics" OR **rwanda** OR ruanda OR **samoa** OR "pacific islands" OR polynesia OR "samoan islands" OR "navigator island" OR "navigator islands" OR **"sao tome and principe"** OR "**saudi arabia**" OR **senegal** OR **serbia** OR **seychelles** OR "**sierra leone**" OR **slovakia** OR "slovak republic" OR **slovenia** OR **melanesia** OR "solomon island" OR "solomon islands" OR "norfolk island" OR "norfolk islands" OR **somalia** OR "**south africa**" OR "**south** **sudan**" OR "**sri lanka**" OR ceylon OR **"saint kitts and nevis"** OR "st. kitts and nevis" OR "**saint lucia**" OR "st. lucia" OR **"saint vincent and the grenadines"** OR "saint vincent" OR "st. vincent" OR grenadines OR **sudan** OR **suriname** OR surinam OR "dutch guiana" OR "netherlands guiana" OR **syria** OR "syrian arab republic" OR **tajikistan** OR tadjikistan OR tadzhikistan OR tadzhik OR **tanzania** OR tanganyika OR **thailand** OR siam OR "**timor leste**" OR "east timor" OR **togo** OR "togolese republic" OR **tonga** OR **"trinidad and tobago"** OR trinidad OR tobago OR **tunisia** OR **turkey** OR **turkmenistan** OR turkmen OR **uganda** OR **ukraine** OR **uruguay** OR **uzbekistan** OR uzbek OR **vanuatu** OR "new hebrides" OR **venezuela** OR **vietnam** OR "viet nam" OR "**middle east**" OR "west bank" OR gaza OR palestine OR **yemen** OR **yugoslavia** OR **zambia** OR **zimbabwe** OR "northern rhodesia" OR "global south" OR "**africa south of the sahara**" OR "sub-saharan africa" OR "subsaharan africa" OR "**africa, central**" OR "central africa" OR "**africa, northern**" OR "north africa" OR "northern africa" OR magreb OR maghrib OR sahara OR "**africa, southern**" OR "southern africa" OR "**africa, eastern**" OR "east africa" OR "eastern africa" OR "**africa, western**" OR "west africa" OR "western africa" OR "**west indies**" OR "**indian ocean islands**" OR **caribbean** OR "**central america**" OR "**latin america**" OR "south and central america" OR "**south america**" OR "**asia, central**" OR "central asia" OR "**asia, northern**" OR "north asia" OR "northern asia" OR "**asia, southeastern**" OR "southeastern asia" OR "south eastern asia" OR "southeast asia" OR "south east asia" OR "**asia, western**" OR "western asia" OR "**europe, eastern**" OR "east europe" OR "eastern europe" OR "developing country" OR "**developing countries**" OR "developing nation$" OR "developing population$" OR "developing world" OR "less developed countr*" OR "less developed nation$" OR "less developed population$" OR "less developed world" OR "lesser developed countr*" OR "lesser developed nation$" OR "lesser developed population$" OR "lesser developed world" OR "under developed countr*" OR "under developed nation$" OR "under developed population$" OR "under developed world" OR "underdeveloped countr*" OR "underdeveloped nation$" OR "underdeveloped population$" OR "underdeveloped world" OR "middle income countr*" OR "middle income nation$" OR "middle income population$" OR "low income countr*" OR "low income nation$" OR "low income population$" OR "lower income countr*" OR "lower income nation$" OR "lower income population$" OR "underserved countr*" OR "underserved nation$" OR "underserved population$" OR "underserved world" OR "under served countr*" OR "under served nation$" OR "under served population$" OR "under served world" OR "deprived countr*" OR "deprived nation$" OR "deprived population$" OR "deprived world" OR "poor countr*" OR "poor nation$" OR "poor population$" OR "poor world" OR "poorer countr*" OR "poorer nation$" OR "poorer population$" OR "poorer world" OR "developing econom*" OR "less developed econom*" OR "lesser developed econom*" OR "under developed econom*" OR "underdeveloped econom*" OR "middle income econom*" OR "low income econom*" OR "lower income econom*" OR "low gdp" OR "low gnp" OR "low gross domestic" OR "low gross national" OR "lower gdp" OR "lower gnp" OR "lower gross domestic" OR "lower gross national" OR lmic OR lmics OR "third world" OR "lami countr*" OR "transitional countr*" OR "emerging economies" OR "emerging nation$") | 2714125 |
| 27 | AK=(**afghanistan** OR **albania** OR **algeria** OR "**american samoa**" OR **angola** OR **"antigua and barbuda"** OR antigua OR barbuda OR **argentina** OR **armenia** OR armenian OR **aruba** OR **azerbaijan** OR **bahrain** OR **bangladesh** OR **barbados** OR "**republic of belarus**" OR belarus OR byelarus OR belorussia OR byelorussian OR **belize** OR "british honduras" OR **benin** OR dahomey OR **bhutan** OR **bolivia** OR **"bosnia and herzegovina"** OR Bosnia-Herzegovina OR bosnia OR herzegovina OR **botswana** OR bechuanaland OR **brazil** OR brasil OR **bulgaria** OR "**burkina faso**" OR "burkina fasso" OR "upper volta" OR **burundi** OR urundi OR "**cabo verde**" OR "cape verde" OR **cambodia** OR kampuchea OR "khmer republic" OR **cameroon** OR cameron OR cameroun OR "**central african republic**" OR "ubangi shari" OR **chad** OR **chile** OR **china** OR **colombia** OR **comoros** OR "comoro islands" OR "iles comores" OR mayotte OR "**democratic republic of the congo**" OR "democratic republic congo" OR **congo** OR zaire OR "**costa rica**" OR **"cote d’ivoire"** OR "cote d’ ivoire" OR "cote divoire" OR "cote d ivoire" OR "ivory coast" OR **croatia** OR **cuba** OR **cyprus** OR "**czech republic**" OR czechoslovakia OR **djibouti** OR "french somaliland" OR **dominica** OR "**dominican republic**" OR **ecuador** OR **egypt** OR "united arab republic" OR "**el salvador**" OR "**equatorial guinea**" OR "spanish guinea" OR **eritrea** OR **estonia** OR eswatini OR **swaziland** OR **ethiopia** OR **fiji** OR **gabon** OR "gabonese republic" OR **gambia** OR **"georgia (republic)"** OR georgian OR **ghana** OR "gold coast" OR **gibraltar** OR **greece** OR **grenada** OR **guam** OR **guatemala** OR **guinea** OR "**guinea bissau**" OR **guyana** OR "british guiana" OR **haiti** OR hispaniola OR **honduras** OR **hungary** OR **india** OR **indonesia** OR timor OR **iran** OR **iraq** OR "isle of man" OR **jamaica** OR **jordan** OR **kazakhstan** OR kazakh OR **kenya** OR "**democratic people’s republic of korea"** OR "**republic of korea**" OR "north korea" OR "south korea" OR korea OR **kosovo** OR **kyrgyzstan** OR kirghizia OR kirgizstan OR "kyrgyz republic" OR kirghiz OR **laos** OR "lao pdr" OR "lao people's democratic republic" OR **latvia** OR **lebanon** OR "lebanese republic" OR **lesotho** OR basutoland OR **liberia** OR **libya** OR "libyan arab jamahiriya" OR **lithuania** OR **macau** OR macao OR "**republic of north macedonia**" OR macedonia OR **madagascar** OR "malagasy republic" OR **malawi** OR nyasaland OR **malaysia** OR "malay federation" OR "malaya federation" OR maldives OR "**indian ocean islands**" OR "indian ocean" OR **mali** OR **malta** OR **micronesia** OR "federated states of micronesia" OR kiribati OR "marshall islands" OR nauru OR "northern mariana islands" OR **palau** OR tuvalu OR **mauritania** OR **mauritius** OR **mexico** OR **moldova** OR moldovian OR **mongolia** OR **montenegro** OR **morocco** OR ifni OR **mozambique** OR "portuguese east africa" OR **myanmar** OR burma OR **namibia** OR **nepal** OR "**netherlands antilles**" OR **nicaragua** OR **niger** OR **nigeria** OR **oman** OR muscat OR **pakistan** OR **panama** OR "**papua new guinea**" OR "new guinea" OR **paraguay** OR **peru** OR **philippines** OR philipines OR phillipines OR phillippines OR **poland** OR "polish people's republic" OR **portugal** OR portuguese republic OR "**puerto rico**" OR **romania** OR **russia** OR "russian federation" OR ussr OR "soviet union" OR "union of soviet socialist republics" OR **rwanda** OR ruanda OR **samoa** OR "pacific islands" OR polynesia OR "samoan islands" OR "navigator island" OR "navigator islands" OR **"sao tome and principe"** OR "**saudi arabia**" OR **senegal** OR **serbia** OR **seychelles** OR "**sierra leone**" OR **slovakia** OR "slovak republic" OR **slovenia** OR **melanesia** OR "solomon island" OR "solomon islands" OR "norfolk island" OR "norfolk islands" OR **somalia** OR "**south africa**" OR "**south** **sudan**" OR "**sri lanka**" OR ceylon OR **"saint kitts and nevis"** OR "st. kitts and nevis" OR "**saint lucia**" OR "st. lucia" OR **"saint vincent and the grenadines"** OR "saint vincent" OR "st. vincent" OR grenadines OR **sudan** OR **suriname** OR surinam OR "dutch guiana" OR "netherlands guiana" OR **syria** OR "syrian arab republic" OR **tajikistan** OR tadjikistan OR tadzhikistan OR tadzhik OR **tanzania** OR tanganyika OR **thailand** OR siam OR "**timor leste**" OR "east timor" OR **togo** OR "togolese republic" OR **tonga** OR **"trinidad and tobago"** OR trinidad OR tobago OR **tunisia** OR **turkey** OR **turkmenistan** OR turkmen OR **uganda** OR **ukraine** OR **uruguay** OR **uzbekistan** OR uzbek OR **vanuatu** OR "new hebrides" OR **venezuela** OR **vietnam** OR "viet nam" OR "**middle east**" OR "west bank" OR gaza OR palestine OR **yemen** OR **yugoslavia** OR **zambia** OR **zimbabwe** OR "northern rhodesia" OR "global south" OR "**africa south of the sahara**" OR "sub-saharan africa" OR "subsaharan africa" OR "**africa, central**" OR "central africa" OR "**africa, northern**" OR "north africa" OR "northern africa" OR magreb OR maghrib OR sahara OR "**africa, southern**" OR "southern africa" OR "**africa, eastern**" OR "east africa" OR "eastern africa" OR "**africa, western**" OR "west africa" OR "western africa" OR "**west indies**" OR "**indian ocean islands**" OR **caribbean** OR "**central america**" OR "**latin america**" OR "south and central america" OR "**south america**" OR "**asia, central**" OR "central asia" OR "**asia, northern**" OR "north asia" OR "northern asia" OR "**asia, southeastern**" OR "southeastern asia" OR "south eastern asia" OR "southeast asia" OR "south east asia" OR "**asia, western**" OR "western asia" OR "**europe, eastern**" OR "east europe" OR "eastern europe" OR "developing country" OR "**developing countries**" OR "developing nation$" OR "developing population$" OR "developing world" OR "less developed countr*" OR "less developed nation$" OR "less developed population$" OR "less developed world" OR "lesser developed countr*" OR "lesser developed nation$" OR "lesser developed population$" OR "lesser developed world" OR "under developed countr*" OR "under developed nation$" OR "under developed population$" OR "under developed world" OR "underdeveloped countr*" OR "underdeveloped nation$" OR "underdeveloped population$" OR "underdeveloped world" OR "middle income countr*" OR "middle income nation$" OR "middle income population$" OR "low income countr*" OR "low income nation$" OR "low income population$" OR "lower income countr*" OR "lower income nation$" OR "lower income population$" OR "underserved countr*" OR "underserved nation$" OR "underserved population$" OR "underserved world" OR "under served countr*" OR "under served nation$" OR "under served population$" OR "under served world" OR "deprived countr*" OR "deprived nation$" OR "deprived population$" OR "deprived world" OR "poor countr*" OR "poor nation$" OR "poor population$" OR "poor world" OR "poorer countr*" OR "poorer nation$" OR "poorer population$" OR "poorer world" OR "developing econom*" OR "less developed econom*" OR "lesser developed econom*" OR "under developed econom*" OR "underdeveloped econom*" OR "middle income econom*" OR "low income econom*" OR "lower income econom*" OR "low gdp" OR "low gnp" OR "low gross domestic" OR "low gross national" OR "lower gdp" OR "lower gnp" OR "lower gross domestic" OR "lower gross national" OR lmic OR lmics OR "third world" OR "lami countr*" OR "transitional countr*" OR "emerging economies" OR "emerging nation$") | 721380 |
| 28 | #25 OR #26 OR #27 | 4008739 |
| 29 | #3 AND #24 AND #28 | 1343 |
|  |  |  |
